# Tbx2 mediates dorsal patterning and germ layer suppression through inhibition of BMP/GDF and Activin/Nodal signaling

**DOI:** 10.1186/s12860-020-00282-1

**Published:** 2020-05-28

**Authors:** Shoshana Reich, Peter Kayastha, Sushma Teegala, Daniel C. Weinstein

**Affiliations:** 1 The Graduate Center, The City University of New York, New York, NY 10016 USA; 2 Department of Biology, Queens College, The City University of New York, Queens, NY 11367 USA

**Keywords:** *Xenopus*, Development, Gastrulation, Ectoderm, Gene regulation, Tbx2, Smads

## Abstract

**Background:**

Members of the T-box family of DNA-binding proteins play a prominent role in the differentiation of the three primary germ layers. VegT, Brachyury, and Eomesodermin function as transcriptional activators and, in addition to directly activating the transcription of endoderm- and mesoderm-specific genes, serve as regulators of growth factor signaling during induction of these germ layers. In contrast, the T-box gene, *tbx2,* is expressed in the embryonic ectoderm, where Tbx2 functions as a transcriptional repressor and inhibits mesendodermal differentiation by the TGFβ ligand Activin. Tbx2 misexpression also promotes dorsal ectodermal fate via inhibition of the BMP branch of the TGFβ signaling network.

**Results:**

Here, we report a physical association between Tbx2 and both Smad1 and Smad2, mediators of BMP and Activin/Nodal signaling, respectively. We perform structure/function analysis of Tbx2 to elucidate the roles of both Tbx2-Smad interaction and Tbx2 DNA-binding in germ layer suppression.

**Conclusion:**

Our studies demonstrate that Tbx2 associates with intracellular mediators of the Activin/Nodal and BMP/GDF pathways. We identify a novel repressor domain within Tbx2, and have determined that Tbx2 DNA-binding activity is required for repression of TGFβ signaling. Finally, our data also point to overlapping yet distinct mechanisms for Tbx2-mediated repression of Activin/Nodal and BMP/GDF signaling.

## Background

Understanding the development of an embryo from a single totipotent cell to a highly differentiated, multicellular organism is a foundational concern of developmental biology. In triploblasts, three embryonic germ layers, ectoderm, mesoderm, and endoderm, give rise to virtually all tissue types. The endodermal germ layer differentiates into pancreas, liver, lung, and other components of the digestive and respiratory systems. The mesodermal layer gives rise the muscular, circulatory, and skeletal systems, and the epidermis, neural tissue, neural crest, and cranial sensory placodes derive from ectoderm. The precise coordination of germ layer differentiation is, by definition, crucial for normal embryogenesis.

During vertebrate development, the formation and patterning of the three germ layers is a rapid and tightly regulated process; studies in the frog *Xenopus laevis* have been essential for our understanding of these processes. An initiating step in development of the germ layers occurs when VegT, a maternally supplied transcription factor, directly initiates an endoderm-specific gene expression program among cells located in the vegetal pole [1]. VegT also activates *nodal* and *nodal*-*related* gene expression; these transcripts encode proteins that induce cells in the region adjacent to the vegetal pole, in the so-called marginal zone, to differentiate into mesoderm [2, 3]. In this VegT-centric model of germ layer formation, differentiation in the animal pole is the consequence of an absence of both extracellular signaling and germ layer-specific transcriptional activation -- VegT is not expressed in this region, and it is far from the source of Nodal signaling; thus, neither endodermal nor mesodermal differentiation ensues, and ectoderm forms in the animal pole “by default” [4, 5].

Recently, however, it has become clear that the suppression of inappropriate cell fate also plays a critical role in germ layer determination in the vertebrate embryo [6]. Several proteins have been implicated in mesendodermal suppression in the ectoderm. For example, *coco* (*dand5)*, a maternally supplied transcript, encodes an inhibitor of TGFβ signaling [7, 8]. Depletion of Coco in the animal cap explant results in ectopic mesodermal formation [7]. Another protein identified as a suppressor of mesendoderm is the Foxi-class transcription factor Xema/Foxi1e. Knockdown of *foxi1e* in animal cap explants leads to an increase in the expression of mesendodermal markers [9]. Moreover, ectopic expression of Foxi1e in the marginal zone inhibits mesoderm development. Foxi1e is a transcriptional activator, and is thus not expected to directly repress mesendodermal gene expression; instead, Foxi1e likely stimulates expression of genes encoding repressor protein(s) that is/are responsible for suppressing ectopic mesendoderm in the animal pole [9]. We recently identified *tbx2*, encoding the repressor T-box protein Tbx2, as one such candidate target of Foxi1e [10].

T-box proteins are an evolutionarily conserved family of transcription factors critical for a number of processes during development [11]. The first identified T-box protein, T, encoded by *brachyury*, induces mesoderm in the early mammalian embryo [12]. In the mouse, a homozygous loss-of-function mutation in *brachyury* is embryonically lethal, highlighting the importance of T-box proteins in early development [13]. T-box proteins have also been implicated in development of craniofacial tissue, liver, heart, and lung [3, 14–17].

The T-box proteins are comprised of five subfamilies; all contain a highly conserved region of 180–200 amino acids, called the T-box, which confers DNA binding specificity [18, 19]. Many T-box proteins have been shown to bind the core T-box binding element TCACACCT [19]. T-box proteins bind to DNA as either monomers or dimers, depending on the protein, and can function as activators or repressors of transcription [20]. Activator and repressor domains have been identified in the carboxyl (C)- or amino (N)- termini of T-box proteins [21, 22].

Two well-studied T-box proteins, VegT and Brachyury, are necessary for formation and patterning of endoderm and mesoderm, respectively; both function as transcriptional activators in this context [3, 23]. Our lab has recently shown that Tbx2 functions in the ectoderm as a transcriptional repressor during gastrulation [10]. When *tbx2* is knocked down in the presumptive ectoderm, ectopic mesendoderm forms; moreover, ectopic Tbx2 suppresses mesendoderm in animal cap explants exposed to Activin or basic Fibroblast Growth Factor (bFGF) [10]. To date, however, little is known about the mechanisms through which Tbx2 promotes repressor activity.

The population of cells in the ectoderm develops into several distinct tissue types. Ventral ectoderm differentiates into epidermis, while the dorsal ectoderm gives rise to neural tissue [24]; cells at the border of these two populations develop into the sensory placodes and neural crest [25, 26]. Bone Morphogenetic Protein-4 (BMP-4), expressed throughout the ectoderm, ventralizes the ectoderm, which subsequently differentiates into epidermis [27]. Dorsally, at the initiation of gastrulation, the Spemann organizer secretes BMP antagonists allowing proximal ectodermal cells to adopt a dorsal, neural fate [27]. Tbx2 misexpression also dorsalizes ectoderm, resulting in neuralization; this effect is accompanied by downregulation of Bone Morphogenetic Protein (BMP) activity [10].

Tbx2 thus appears to suppress signaling through both the Activin/Nodal and BMP/GDF branches of the TGFβ pathway. In canonical TGFβ signaling, upon ligand binding, the TGFβ type II receptor phosphorylates the TGFβ type I receptor, which propagates the signal and phosphorylates the carboxyl terminus of the intracellular signal transducers Smad1/5 or Smad2/3, downstream of BMP/GDF or Activin/Nodal ligands, respectively [28, 29]. These receptor-mediated Smads then form a heteromeric complex with Smad4 which translocates to the nucleus where it associates with transcription factors to regulate gene expression [28].

The discovery that Tbx2 has a global impact on TGFβ signaling prompted us to examine more directly the relationship between Tbx2 and components of the Activin/Nodal and BMP/GDF networks. Here we report that Tbx2 physically associates with both Smad1 and Smad2, essential intracellular mediators of BMP and Nodal signaling, respectively. Deletion analysis of the Tbx2 protein identified the T-box DNA-binding domain as sufficient for Tbx2-Smad binding. Structure-function analyses suggest a model in which Tbx2 regulates interaction of Smads and Smad-interacting proteins in the nucleus.

## Results

### Tbx2 physically associates with multiple Smad proteins

**Fig. 1:**
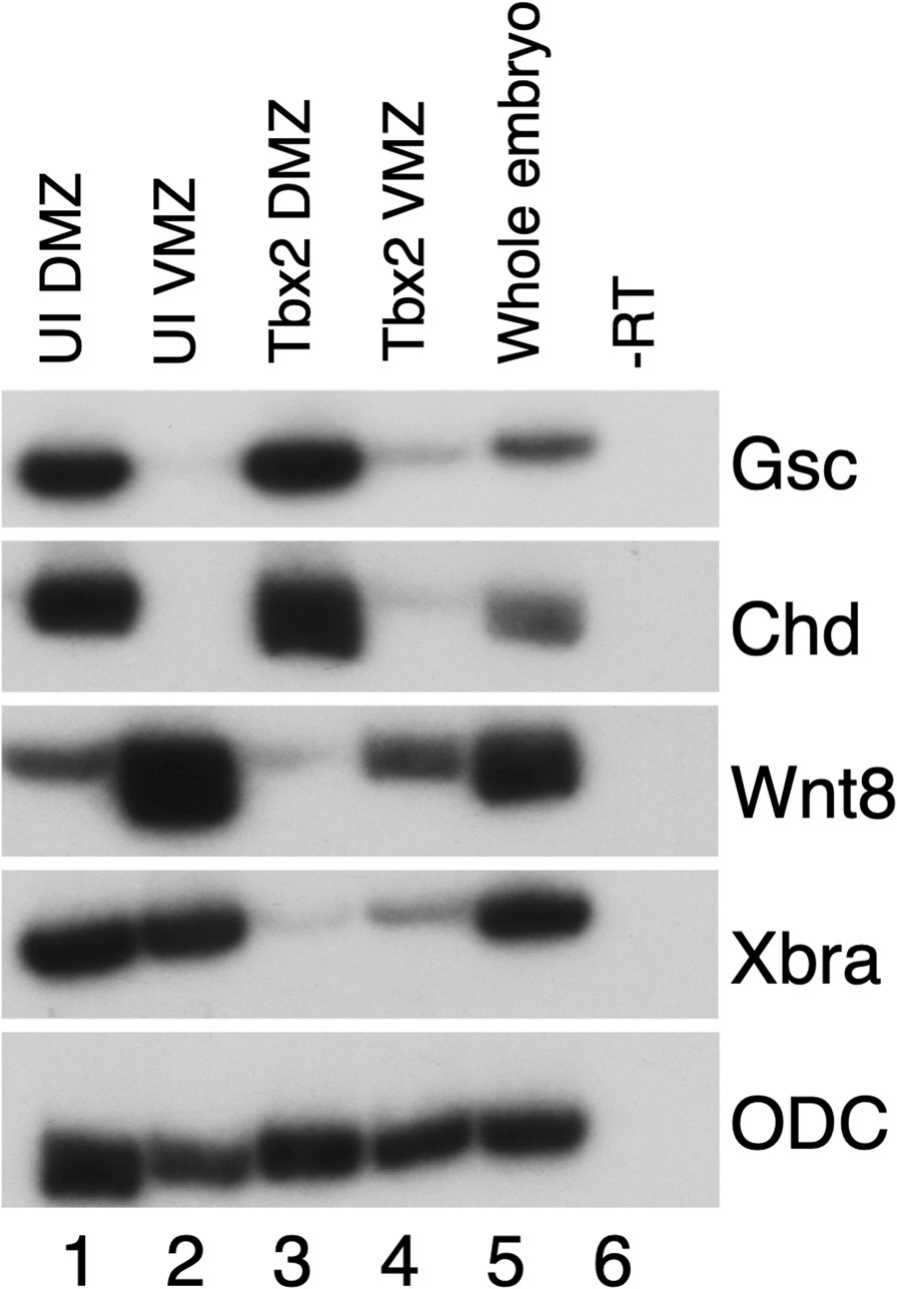
Tbx2 dorsalizes ventral mesoderm. RT-PCR analysis of marginal zone explants. Embryos were injected radially at early cleavage stages with 1 ng of *tbx2*; marginal zone explants were dissected at stage 10

We have previously reported that Tbx2 inhibits mesoderm induction in animal cap explants treated with either basic Fibroblast Growth Factor (bFGF) or the TGFβ ligand Activin [10]. We find that misexpression of Tbx2 also inhibits expression of the panmesodermal marker *brachyury* in ventral and dorsal marginal zone explants, indicating that Tbx2 represses mesoderm formation in the context of the embryo (Fig. 1) [30]. Notably, ventral marginal zone explants show an increase in the dorsal markers *chordin* and *goosecoid* and repression of the ventral marker *wnt8*, demonstrating that Tbx2 has a dorsalizing effect on the mesoderm, as well as the ectoderm [10, 31–33] (Fig. 1). These and earlier published studies suggest that Tbx2 inhibits both Activin/Nodal and BMP/GDF signaling in the early embryo [10]. This raised the prospect that Tbx2 may inhibit TGFβ signaling via direct interaction with TGFβ signaling transducers.

**Fig. 2:**
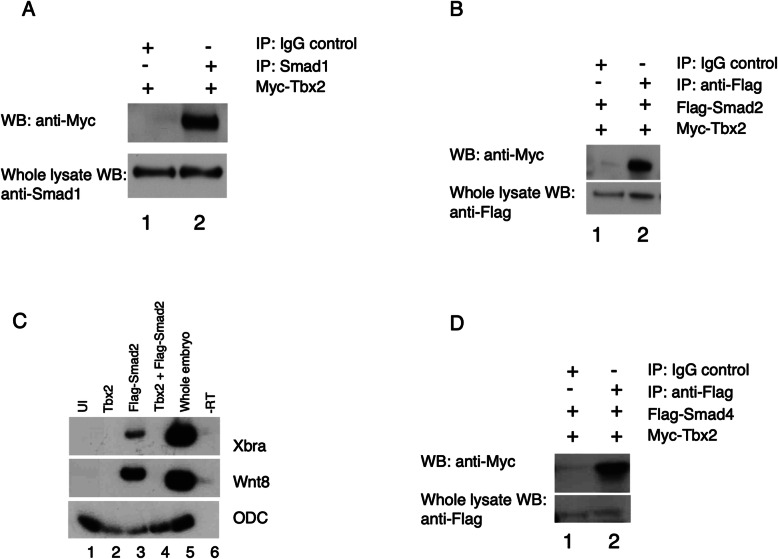
Tbx2 associates with R-Smads **a**). Tbx2 physically associates with Smad1. 1 ng *myc-tbx2* RNA was injected at early cleavage stages. Pull-down of native Smad1 from injected embryos leads to co-immunoprecipitation of exogenous Tbx2. Normal rabbit IgG antibodies were used in parallel studies as a negative control. **b** Tbx2 physically associates with Smad2. 1 ng of *myc-tbx2* and 1 ng of *flag-smad2* were injected at early cleavage stages. Pull-down of Flag-Smad2 from injected embryos leads to co-immunoprecipitation of exogenous Tbx2. Normal rabbit IgG antibodies were used in parallel studies as a negative control **c**) Tbx2 represses Smad2-mediated mesoderm induction. Embryos were injected with *tbx2* (1 ng), *flag-smad2* (1 ng)*,* or *tbx2* (1 ng) and *flag-smad2* (1 ng) at the two-cell stage and animal caps were explanted at stage 8.5. **d** Tbx2 physically associates with Smad4. 1 ng of *myc-tbx2* and 1 ng of *flag-smad4* were injected at early cleavage stages. Pull-down of Flag-Smad4 from injected embryos leads to co-immunoprecipitation of of exogenous Tbx2. Normal rabbit IgG antibodies were used in parallel studies as a negative control

To address this possibility of direct interaction between Tbx2 and intracellular components of the TGFβ signaling network, we first examined whether Tbx2 physically associates with the Smad1 and Smad2 proteins, branch-specific mediators of BMP/GDF and Activin/Nodal signaling, respectively. As we have not been able to obtain an antibody that recognizes native Tbx2, we generated a Myc-epitope-tagged Tbx2 construct (Myc-Tbx2), and injected *myc-tbx2* synthetic RNA into early cleavage stage embryos. Immunoprecipitation and Western blot analysis demonstrated that native Smad1 physically associates with exogenous Tbx2 in *Xenopus* embryos (Fig. 2a). We have not been able to detect native *Xenopus* Smad2 following immunoprecipitation using commercially-available antibodies; thus, we next co-injected *myc-tbx2* and *flag-smad2* synthetic RNA into early cleavage stage embryos. Physical association between exogenous Smad2 and Tbx2 in *Xenopus laevis* embryos was observed (Fig. 2b). Tbx2 inhibits mesodermal marker gene expression stimulated by Smad2, consistent with a physiological role for Tbx2-Smad2 interaction during germ layer differentiation (Fig. 2c).

Following pathway-specific activation, both branches of the TGFβ pathway converge on the “co-Smad” Smad4, which associates with C-terminally phosphorylated forms of both Smad1 and Smad2 [34]. As with Smad1 and Smad2, exogenous Tbx2 physically associates with Flag epitope-tagged human Smad4 (Fig. 2d). Taken together, these studies demonstrate that Tbx2 associates with both pathway-specific and pathway-shared Smad proteins. Additionally, these data indicate that Tbx2 is unlikely to inhibit TGFβ signaling via sequestration of R-Smads from Smad4.

### Identification of the Tbx2 domain sufficient for R-Smad association

**Fig. 3:**
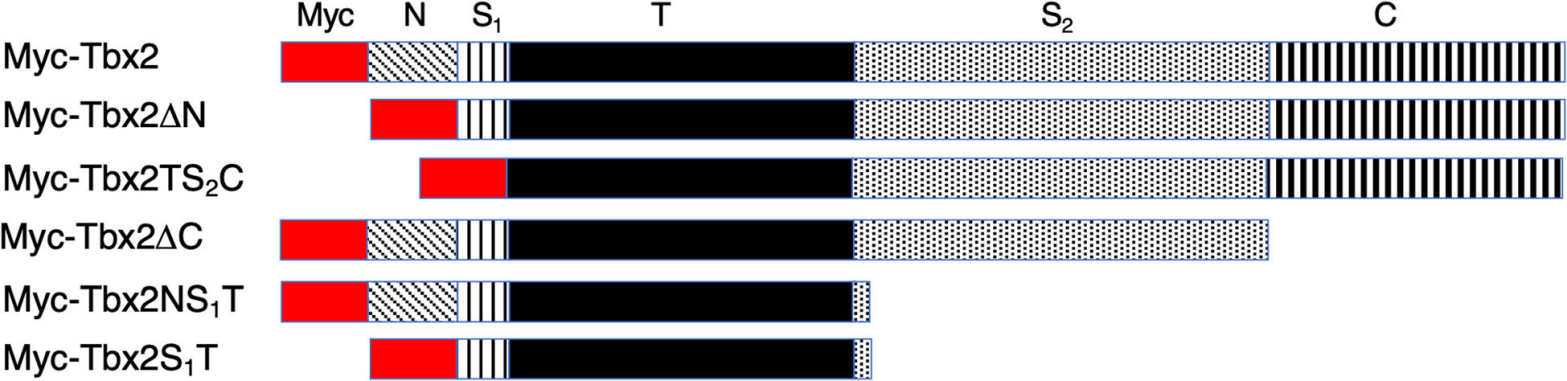
Depictions of Myc-tagged Tbx2 deletion constructs. **“**Myc” refers to a 6x Myc epitope tag. N and C refer to the N and C termini, respectively. S_1_ and S_2_ refer to the first and second spacer, respectively

**Fig. 4:**
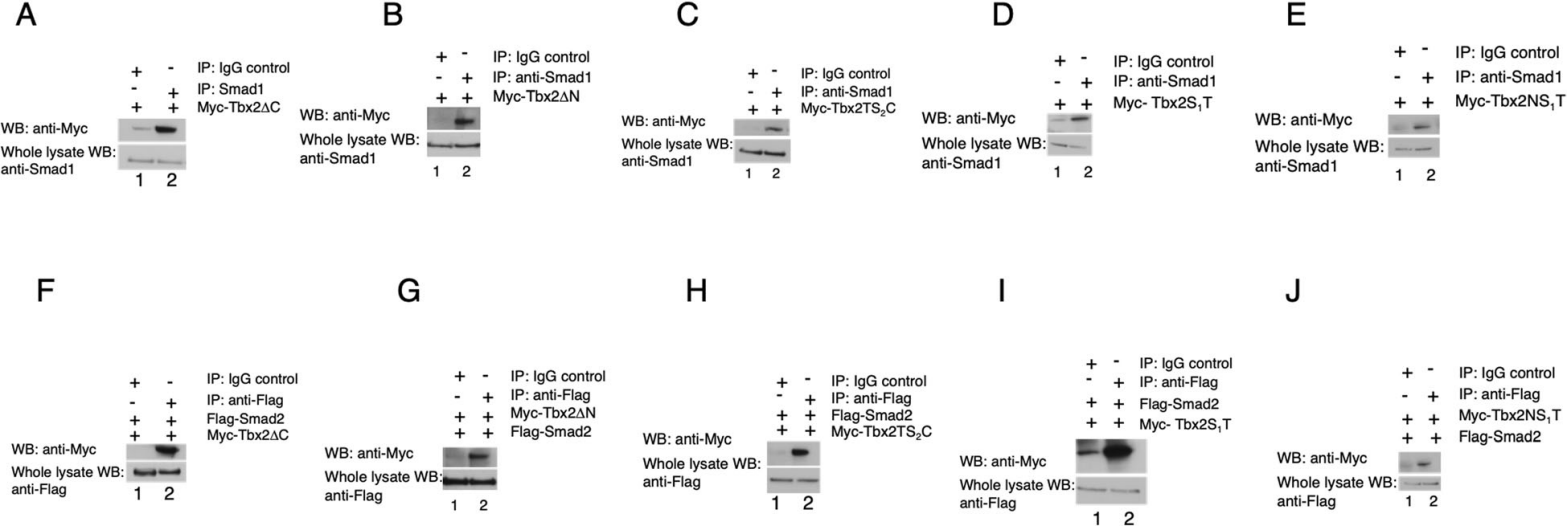
The Tbx2 Spacer 1 and T-box domains are sufficient for R-Smad association. **a-e** Smad1 physically associates with Tbx2 via the T-box. Embryos were injected at the two-cell stage with 1 ng of RNA synthesized from the indicated deletion construct. Pull-down of native Smad1 from injected embryos leads to co-immunoprecipitation of the exogenous deletion construct. Normal rabbit IgG antibodies were used in parallel studies as a negative control. **f-j** Smad2 physically associates with Tbx2 via the T-box. Embryos were injected at the two-cell stage with 1 ng of RNA synthesized from the indicated deletion construct and *flag-smad2*. Pull-down of Flag-Smad2 from injected embryos leads to co-immunoprecipitation of the exogenous deletion construct. Normal rabbit IgG antibodies were used in parallel studies as a negative control

Our determination of interactions between Tbx2 and Smad proteins prompted us to ask whether these associations are central to Tbx2’s role in suppression of dorsal and/or extraectodermal fate. To determine whether Tbx2 requires interaction with Smad1 and Smad2 to mediate dorsoventral patterning or mesendodermal suppression, respectively, we first sought to identify the domains of Tbx2 protein required for Smad interaction. Toward this end, we constructed multiple Tbx2 deletion constructs, tagged with a Myc epitope (Fig. 3). These deletion constructs were designed so that each construct lacks one or more domains of Tbx2, with the goal of eliminating the region necessary for R-Smad interaction. Following injection of RNA encoding these constructs, we performed immunoprecipitation assays using either a Smad1 antibody or, after co-injection of *myc-tbx2* and *flag-smad2*, a Flag epitope-specific antibody, followed by SDS-PAGE and Western blotting with an anti-Myc antibody. These studies identify the T-box region as sufficient for association with either Smad1 or Smad2; deletion of the N or C termini had no effect on R-Smad association. Tbx2 constructs with the first or second spacer deleted also associated with both Smad1 and Smad2 ((Fig. 4a-j).

### Activity of deletion constructs

**Fig. 5:**
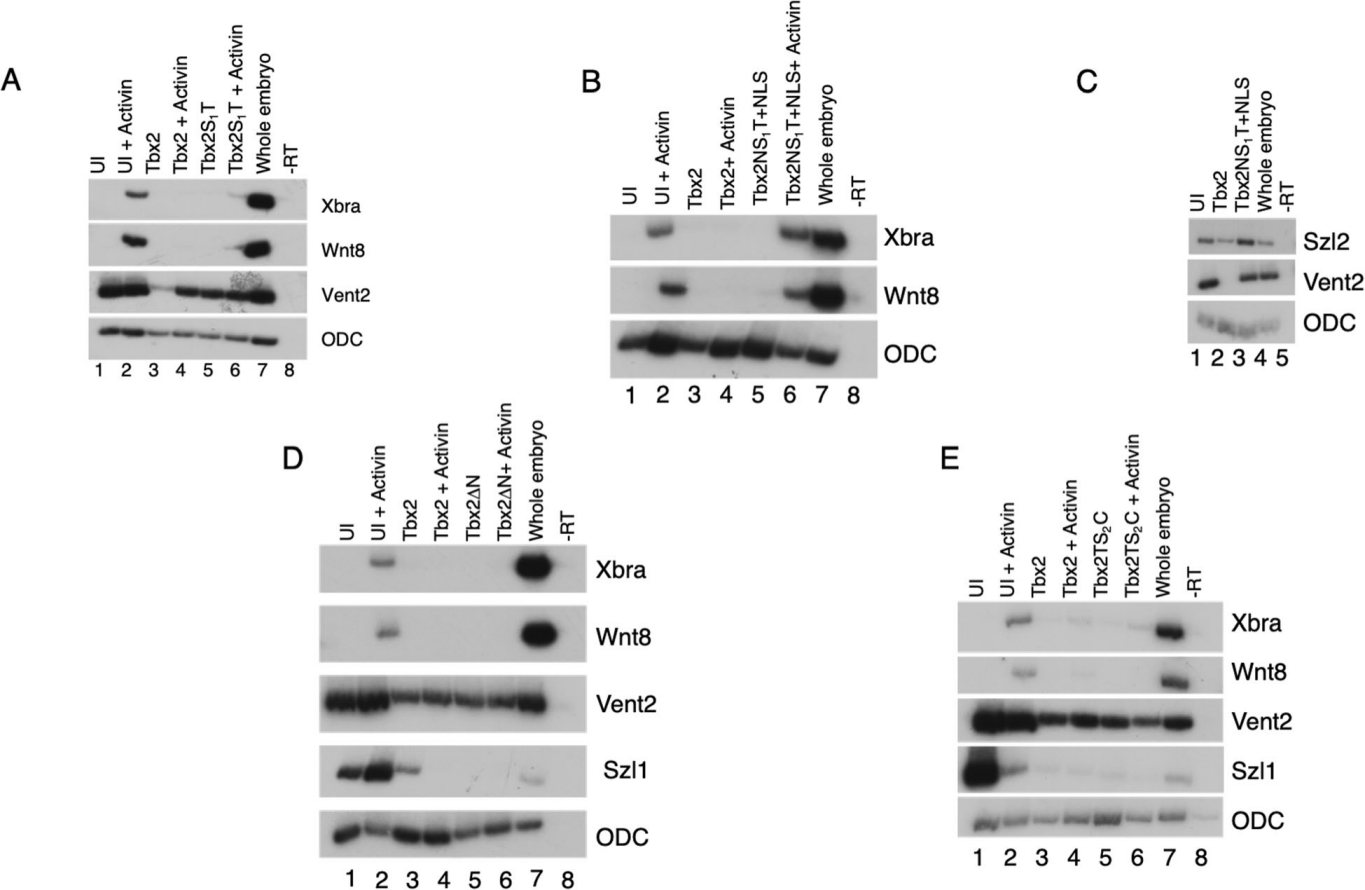
Effects of Tbx2 deletion constructs on the BMP/GDF and Activin/Nodal pathways **a**) Embryos were injected with 2 ng of *tbx2* or *tbx2S*_*1*_*T* in the animal pole at the two-cell stage. Animal caps were explanted at stage 8.5. Activin was added (2.5 ng/ml) as indicated. **b** Embryos were injected with 2 ng of *tbx2* or *tbx2NS*_*1*_*T* in the animal pole at the two-cell stage. Animal caps were explanted at stage 8.5. Activin was added (2.5 ng/ml) as indicated. **c** Embryos were injected with 2 ng of *tbx2* or *tbx2NS*_*1*_*T* in the animal pole at the two-cell stage. Animal caps were explanted at stage 8.5. **d** Embryos were injected with 1 ng *tbx2* or *tbx2*Δ*N* in the animal pole at the two-cell stage. Animal caps were explanted at stage 8.5. Activin was added (2.5 ng/ml) as indicated **e**) Embryos were injected with 1 ng *tbx2* or *tbx2TS*_*2*_C in the animal pole at the two-cell stage. Animal caps were explanted at stage 8.5. Activin was added (2.5 ng/ml) as indicated

Because the region of Smad association is within the T-box, we questioned whether the smallest construct that contained the T-box has activity. We expected this construct, Tbx2S_1_T, to function, if at all, like Tbx2ΔC as both lack a previously identified C-terminal repressor domain. Previous work from our lab showed that Tbx2ΔC does not repress Activin-mediated mesoderm expression [10]. To test the activity of Tbx2S_1_T, we used an embryological assay. Animal caps were explanted at stage 8.5 from embryos injected at the 2-cell stage with RNA transcribed from Tbx2S_1_T and cultured until stage 11 in the presence or absence of Activin. These samples were then assayed for mesodermal gene expression. To our surprise, this construct repressed Smad2/Activin-mediated mesoderm induction in animal caps, suggesting the presence of a second, previously unidentified repressor domain in Tbx2S_1_T (Fig. 5a). Unlike full-length Tbx2, misexpression of this construct did not repress all ventral markers; *vent2 *was not repressed, while repression of *szl* was sometimes observed, indicating that complete ventral repression may require an as-yet undefined composite of domains (Fig. 5a, data not shown). Alternatively, it is possible that a higher concentration of *tbx2S*_*1*_*T* is required for ventral than for mesodermal repression.

This result led us to question why the presumed second Smad2/Activin repressor domain is not active in Tbx2ΔC. We hypothesize that there is a region present in Tbx2ΔC that functions as either an activator domain or an inhibitor of the second repressor domain. To test this possibility, we injected RNA transcribed from the construct missing both the C-terminus and the second spacer, Tbx2NS_1_T, into two-cell stage embryos. Animal caps were explanted at stage 8.5 and cultured in the presence or absence of Activin. Animal caps injected with *tbx2NS*_*1*_*T* RNA did not repress Activin-mediated mesoderm induction or ventral markers (Fig. 5b, c). These data supports our hypothesis, and indicates that the presence of the N-terminus inhibits activity of the repressor located in Tbx2S_1_T. Deletion of only the N-terminus has no effect on Tbx2 activity; animal caps explanted from embryos injected with RNA transcribed from Tbx2ΔN did not express mesodermal markers in the presence of Activin (Fig. 5d). Finally, RNA transcribed from Tbx2TS_2_C, was injected and assayed for activity. This construct functions like the full length Tbx2 in both the Smad1/BMP and Smad2/Activin pathways (Fig. 5e). These results indicate that a construct containing the T-box, second spacer, and C-terminus renders the N-terminus and the first spacer unnecessary for wild-type repression of both the Activin/Nodal and BMP/GDF pathways (Fig. 5e).

### Tbx2 regulation of Smad C-terminal phosphorylation

**Fig. 6:**
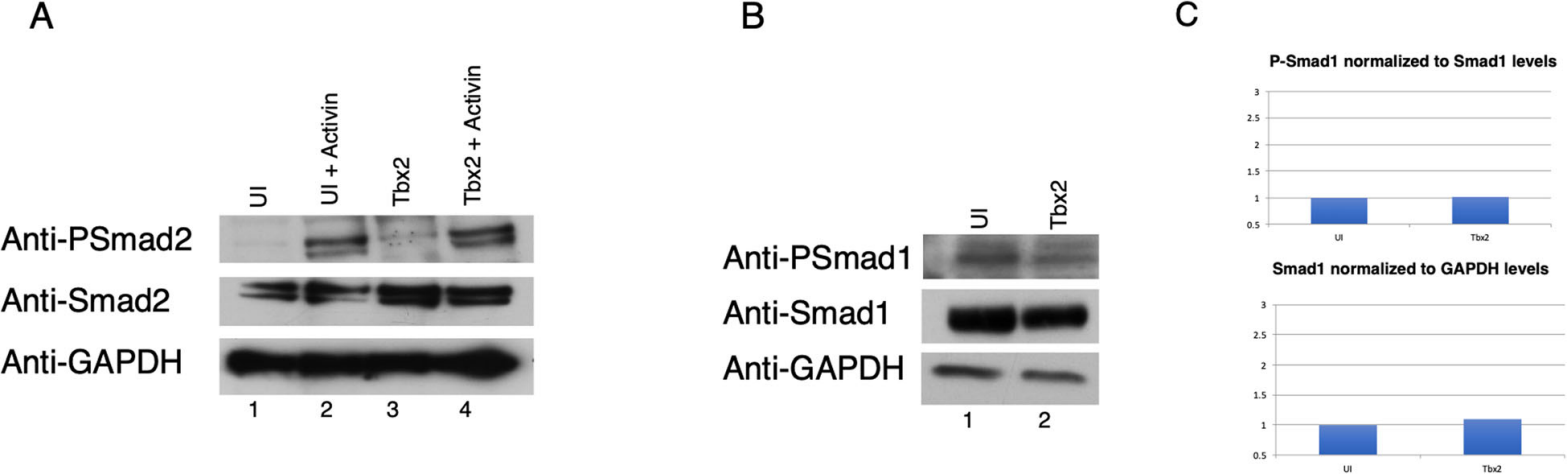
Tbx2 does not decrease levels of C-terminal phosphorylation of Smad1 and Smad2 **a**) Embryos were injected with 1 ng *tbx2* at the two-cell stage; animal caps were explanted at stage 8.5 and incubated in Activin (2.5 ng/ml), as indicated, until late gastrula stages. Uninjected embryos were used in parallel studies. **b** Embryos were injected with 1 ng *tbx2* at the two-cell stage and animal caps were explanted at stage 8.5 and incubated until late gastrula stages. Uninjected embryos were used in parallel studies. **c** Quantification of data shown in B, indicating no change in Smad1 protein levels or phosphorylation of Smad1 as a result of ectopic *tbx2* expression

As mentioned above, TGFβ-induced nuclear accumulation and activation of Smad1 and Smad2 is mediated by phosphorylation of Serine residues at their C-termini [35]. We reasoned that Tbx2 might inhibit Smad activation via inhibition of Smad C-terminal phosphorylation. To test this possibility, animal cap explants derived from embryos injected with *tbx2* RNA and cultured in the presence or absence of Activin were assayed at gastrula stages for embryological activity and for Smad C-terminal phosphorylation by Western blotting (Cell Signaling) [36]. Uninjected embryos were processed in parallel. Notably, we found that Tbx2 does not decrease Smad2 phosphorylation levels in the presence of Activin, indicating that Tbx2 does not inhibit Activin/Nodal signaling through hypophosphorylation of Smad2 C-terminal Serine residues (Fig. 6a). Similar experiments were performed to examine the potential regulation by Tbx2 on Smad1 C-terminal phosphorylation. In these studies, Smad1 C-terminal phosphorylation was similarly unaffected by the presence of ectopic Tbx2 (Fig. 6b, c). These studies demonstrate that Tbx2 does not inhibit TGFβ signaling via C-terminal dephosphorylation of Smad1 or Smad2.

### Tbx2 activity requires arginine 122

**Fig. 7:**
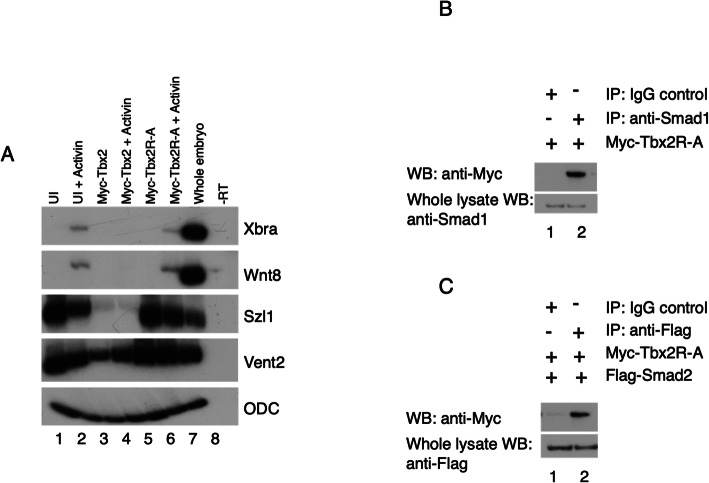
DNA binding is necessary for Tbx2 repressor activity. **a** RT-PCR analysis of animal cap explants injected with Tbx2 DNA binding mutant construct. Embryos were injected with 1 ng *myc-tbx2R-A* or *myc-tbx2* at the two-cell stage; animal caps were explanted at stage 8.5 and incubated in Activin (2.5 ng/ml) where indicated until late gastrula stages. **b** Smad1 physically associates with Tbx2R-A. Embryos were injected at the two-cell stage with 1 ng of RNA synthesized from Myc-Tbx2R-A. Pull-down of native Smad1 from injected embryos leads to co-immunoprecipitation of exogenous Myc-Tbx2R-A. Normal rabbit IgG antibodies was used in parallel studies as a negative control. **c** Smad2 physically associates with Myc-Tbx2R-A. Embryos were injected at the two-cell stage with 1 ng *myc-tbx2R-A* and 1 ng *flag-smad2*. Pull-down of Flag-Smad2 from injected embryos leads to co-immunoprecipitation of exogenous Myc-Tbx2R-A. Normal rabbit IgG antibodies were used in parallel studies as a negative control

The T-box protein Tbx20 mediates repressor activity independent of DNA binding [37]. We therefore questioned whether DNA-binding was necessary for Tbx2 activity. We thus attempted to separate the DNA binding activity of Tbx2 from the physical association of Tbx2 with R-Smads. Previous studies of T-box proteins have shown that Arginine 122 is necessary for Tbx2 DNA binding activity [38]. An Arginine to Alanine mutation in the T-box of Myc-Tbx2 (Myc-Tbx2R-A) abolishes repressor activity in both the BMP/GDF and Activin/Nodal branches of the TGFβ pathway (Fig. 7a). Co-immunoprecipitaion experiments with Myc-tagged Tbx2R-A, Smad1, and Smad2 show that Tbx2 associates with R-Smads even when DNA-binding activity is abolished (Fig. 7b,c). These data indicate that Tbx2 physically associates with R-Smads independent of DNA binding, and that this interaction is not sufficient for repression of TGFβ signaling.

## Discussion

T-box transcription factors mediate cell specification and patterning in the early vertebrate embryo. Our results point to a central role for Tbx2-Smad interactions in the inhibition of both ventral and mesodermal fates. The inability of Tbx2S_1_T to consistently repress ventral markers, while effectively blocking mesoderm induction, indicates that different domains of Tbx2 may be necessary to repress the Activin/Nodal and BMP/GDF pathways. We find that the Tbx2 Spacer 1 and T-box domains are sufficient for interaction with R-Smads. Because the T-box is necessary for Tbx2 activity, it was not possible to inhibit R-Smad association without eliminating a domain presumably required for DNA binding. A point mutation in the T-box of Tbx2 demonstrates that Tbx2 associates with R-Smads independently of DNA binding. Misexpression of the Tbx2 DNA-binding mutant does not repress ventral or mesodermal markers, indicating that repression of ventral and mesodermal markers requires DNA binding.

In a previous study, we demonstrated that Tbx2 dorsalizes ectoderm and is necessary and sufficient for the suppression of mesoderm and endoderm in the animal pole of the gastrula stage *Xenopus laevis* embryo [10]. Like Tbx2, several other repressors of mesodermal and ventral gene expression in the ectoderm function by inhibiting both the Activin/Nodal and BMP/GDF branches of the TGFβ signaling pathway [8, 39, 40]. In the TGFβ signaling pathway, R-Smad proteins are present in the cytoplasm until they are phosphorylated following ligand receptor binding, and translocate into the nucleus [35]. As knockdown of *tbx2* in animal cap explants stimulates expression of mesodermal markers, it is possible that Tbx2 prevents low levels of phosphorylated R-Smads present in the nucleus from having an appreciable effect on mesendodermal and/or ventral gene expression.

Various T-box transcription factors such as Eomesodermin, Brachyury, and Tbx20 have been shown to associate with either Smad1 or Smad2 and have been shown to function in either the BMP/GDF or Activin/Nodal pathways [37, 41, 42]. Here, we describe a T-box protein that associates with both classes of R-Smad and that represses both branches of the TGFβ signaling network. The association between Brachyury and Smad1 requires BMP signaling and is necessary for the activation of Vent2.2 (Xom), a repressor of *goosecoid* [41]. If the association between Brachyury and Smad1 is attenuated, Brachyury activates *goosecoid*, and only high levels of both Brachyury and the BMP antagonist Noggin can activate *goosecoid* [41]. These results suggest that Brachyury activity is dependent on the level of BMP signaling, and subsequently, the levels of Smad1 in the nucleus. An analogous scenario possibly occurs in the presumptive ectoderm, whereby ventral Tbx2 activity is suppressed by high levels of nuclear Smad1.

The domains of Smad1 and Smad2 necessary for Tbx2 association remain unknown. Brachyury, Eomesodermin, and Tbx20 have all been shown to physically associate with R-Smads [37, 41, 42]. To our knowledge, the R-Smad domain(s) necessary for the association with Eomesodermin and Tbx20 are not defined. Brachyury associates via its N-terminus with Smad1 via the Smad1 C-terminus [41]. Sequence analysis does not show any appreciable alignment between the N-terminus of Brachyury and any region of Tbx2. Additionally, unlike Tbx2, Brachyury does not associate with Smad2 [41].

T-box proteins have been shown to play an important role in a neuromesodermal bimodal “switch” to either promote mesodermal or neural gene expression at the expense of the other [43]. The current study and previous data from our group show that Tbx2 promotes neuralization at the expense of a mesendodermal fate [10]. Our data suggest that Tbx2 may not inhibit R-Smad function directly; it is also possible that the Tbx2-Smad association is necessary for Tbx2 repressor activity in the context of germ layer differentiation. Many transcription factors that are responsible for controlling cell fate commitment in developing embryos also regulate development of pluripotent stem cells [44, 45]. Identifying transcription factors necessary for germ layer differentiation, including Tbx2, and defining their mechanisms of action thus has far-reaching implications in multiple areas of biology.

## Conclusion

During gastrulation, T-box transcription factors play important roles in cell fate specification [2, 3, 10, 30]. For the first time, we show physical association between Tbx2 and intracellular mediators of both the BMP/GDF and Activin/Nodal signaling pathways. Protein-protein interaction assays indicate that the Tbx2 T-box is sufficient for association with R-Smads; furthermore, structure/function analysis identified a novel repressor domain in Tbx2 located in either the first spacer or the T-box. Inhibition of DNA binding by Tbx2 blocks its ability to inhibit ventral and mesodermal fate. Our studies suggest a model in which Tbx2 physically associates with Smad1 or Smad2, represses transcription of BMP/GDF or Activin/Nodal targets, and thus inhibits expression of ventral and mesodermal genes, respectively.

## Methods

### RNA preparation, explant dissection, and embryo culture

RNA was synthesized in vitro in the presence of cap analog using the mMessage mMachine kit (Ambion). Microinjection, explant dissection, and embryo culture were performed as described [3, 14–17]. All constructs were injected at a concentration of 1 ng/embryo except in the experiments with *tbx2NS*_*1*_*T.* In this series of experiments, embryos were injected with 2 ng/embryo of *tbx2NS*_*1*_*T* or 2 ng of *tbx2* (1 ng of RNA injected into each cell at the two-cell stage). For all other experiments, where the constructs were injected with a total of 1 ng/embryo, embryos injected at the two-cell stage were injected with 500 pg into each cell. For all animal cap and marginal zone explant experiments, a minimum of five explants was used for each condition, and each experiment was performed at least three times; results were consistent between all trials.

### Construct preparation

To generate Myc-Tbx2ΔC, six Myc epitope tags were added upstream of a Tbx2ΔC construct, the latter containing amino acids 1-518 of Tbx2 [10, 46]. To generate Tbx2ΔN, amino acids 54-688 were subcloned into pCS2++. For the Myc-Tbx2ΔN, DNA encoding amino acids 54-688 were subcloned into pCS2MT (constructed by D. L. Turner and R. A. W. Rupp). For Tbx2NS_1_T, amino acids 290-688 were deleted from Tbx2 in pCS2+. The same amino acids were deleted from Myc-Tbx2 to generate Myc-Tbx2NS_1_T (Genewiz). In this construct and all subsequent constructs with the Tbx2 T-box at the C terminus, ten amino acids from spacer 2 were added to include the nuclear localization sequence [47]. To 16 generate Tbx2TS_2_C, amino acids 1-54 were deleted from Tbx2 in pCS2+. The same amino acids were deleted in Myc-Tbx2 to generate Myc-Tbx2TS_2_C (Genewiz). To construct Tbx2S_1_T and Myc-Tbx2S_1_T, amino acids 290-688 were deleted from Tbx2ΔN and MycTbx2ΔN, respectively. Myc-Tbx2R-A was generated by a single amino acid mutation of Arginine 122 to Alanine (AGG to GCT) in Myc-Tbx2 (Genewiz) [38]. Flag-Smad2 was purchased from Addgene (plasmid #14042). Flag-Smad4 was a gift from Dr. Gerald Thomsen [48].

### Reverse transcription-polymerase chain reaction (RT-PCR)

*Xenopus laevis* embryos were staged according to Nieuwkoop and Faber, 1967 and harvested at appropriate stages according to morphological criteria. RNA was prepared using RNA Bee RNA isolation reagent (Tel-Test Inc.). RT-PCR was performed as described (Wilson and Hemmati-Brivanlou, 1995). All primer sequences are as described: *ODC*, *Xbra*, *Wnt8, chordin, and goosecoid* [9]; *Sizzled1* [49]; *Sizzled2* [50]; *Bmp4* [51]; *Xvent2* [52].

### Western blot analysis

Western blot analysis was performed largely as described [53]. Animal caps (10–15 per condition) were lysed in 5ul/animal cap of lysis buffer (150 mM NaCl, 20 mM Tris pH 7.5, 1% Nonidet P-40, 1 mM EDTA, and 1 protease and phosphatase inhibitor tablet/10 ml of buffer) (Thermofisher) [53]. After incubation on ice for 30 min, animal cap lysates were centrifuged at 4 °C for 5 min at 14,000 g. Clear supernatant was retained. 5–15 ul of supernatant was run for each sample. Antibodies against phospho-Smad1/5 (S463/465) (Cell Signaling Technology), Smad1 (Cell Signaling Technology), Phospho-Smad2 (S465/467) (Cell signaling technology), Smad2/3 (Cell signaling technology) and GAPDH (Sigma) were all used at 1:1000 dilution. Secondary antibodies (donkey anti-rabbit IgG, or donkey anti-mouse IgG) coupled to horseradish peroxidase (Jackson ImmunoResearch) were used at 1:10,000 dilution. Bands were subjected to densitometric analysis and graphed.

### Co-immunoprecipitation

RNA from all Myc-tagged constructs was injected into early cleavage stages embryos. Injected embryos were harvested at late gastrula stages. Co-immunoprecipitation experiments were performed largely as described [53]. Embryos were lysed in 10ul/embryo of lysis buffer (150 mM NaCl, 20 mM Tris pH 7.5, 1% Nonidet P-40, 1 mM EDTA, and 1 protease inhibitor tablet/10 ml of buffer) (Thermofisher) [53]. After incubation on ice for 30 min, embryo lysates were centrifuged at 4 °C for 5 min at 14,000 g. Clear supernatant was retained and then split equally into control and experimental samples. 10 ul of clear supernatant was removed as “input” from each sample. Embryo lysates were incubated with rotation overnight at 4 °C with either anti-Smad1 (333 μg/ml) or anti-Flag **(**800 μg/ml) antibodies at 1:150 dilution or normal Rabbit IgG (1 mg/ml) with the IgG normalized to experimental samples (Cell Signaling Technology), followed by incubation with Dynabeads Protein G (Novex) with rotation at 4 °C for one hour. Samples were washed 6 times with 200 ul of lysis buffer and eluted with 20ul of 1X NuPage LDS sample buffer and 5ul of .5 M dithiothreitol. The elution was subject to SDS-PAGE. Antibodies against Myc (Sigma) were used to probe the blot at 1:1000 dilution. Secondary antibodies (donkey anti-mouse IgG) coupled to horseradish peroxidase (Jackson ImmunoResearch) were used at 1:10,000 dilution.

## Supplementary information

**Additional file 1:** Figure 1- unprocessed data RT-PCR gels shown in figure 1

**Additional file 2:** Figure 2A- unprocessed data. Western blots shown in Figure 2A. Figure 2B- unprocessed data Western blots shown in 2B. Figure 2C- unprocessed data RT-PCR gel shown in Figure 2C. Figure 2D- unprocessed data Western blots shown in Figure 2D.

**Additional file 3:** Figure 4A- unprocessed data. Western blots shown in figure 4A. Figure 4B- unprocessed data Western blots shown in figure 4B. Figure 4C- unprocessed data Western blots shown in figure 4C. Figure 4D- unprocessed data Western blots shown in figure 4D. Figure 4E- unprocessed data Western blots shown in figure 4E. Figure 4F- unprocessed data Western blots shown in figure 4F. Figure 4G- unprocessed data Western blots shown in figure 4G. Figure 4H- unprocessed data Western blots shown in figure 4H. Figure 4I- unprocessed data Western blots shown in figure 4I. Figure 4J- unprocessed data Western blots shown in figure 4J

**Additional file 4:** Figure 5A- unprocessed data. RT-PCR gels shown in figure 5A. Figure 5B- unprocessed data RT-PCR gels shown in figure 5B. Figure 5C- unprocessed data. RT-PCR gels shown in figure 5C. Figure 5D- unprocessed data. RT-PCR gels shown in figure 5D. Figure 5E- unprocessed data. RT-PCR gels shown in figure 5E

**Additional file 5:** Figure 6A- unprocessed data. Western blots shown in figure 6A. Figure 6B- unprocessed data Western blots shown in figure 6B

**Additional file 6:** Figure 7A- unprocessed data. RT-PCR gels shown in figure 7A. Figure 7B- unprocessed data Western blots shown in figure 7B. Figure 7C- unprocessed data Western blots shown in figure 7C

## Data Availability

All original gels and blots have been uploaded as Additional File 1, Additional File 2A, Additional File 2B, Additional File 2C, Additional File 2D, Additional File 3A, Additional File 3B, Additional File 3C, Additional File 3D, Additional File 3E, Additional File 3F, Additional File 3G, Additional File 3H, Additional File 3I, Additional File 3 J, Additional File 4A, Additional File 4B, Additional File 4C, Additional File 4D, Additional File 4E, Additional File 5A, Additional File 5B, Additional File 6A, Additional File 6B, and Additional File 6C.

## Abbreviations

TGFβ: Transforming growth factor beta
BMP: Bone morphogenetic protein
GDF: Growth differentiation factor
