# Supplementary figures and images for: Tbx2 mediates dorsal patterning and germ layer suppression through inhibition of BMP/GDF and Activin/Nodal signaling
Source: BMC Mol Cell Biol. 2020 May 28;21:39. doi: 10.1186/s12860-020-00282-1 (PMC7257154; doi:10.1186/s12860-020-00282-1)

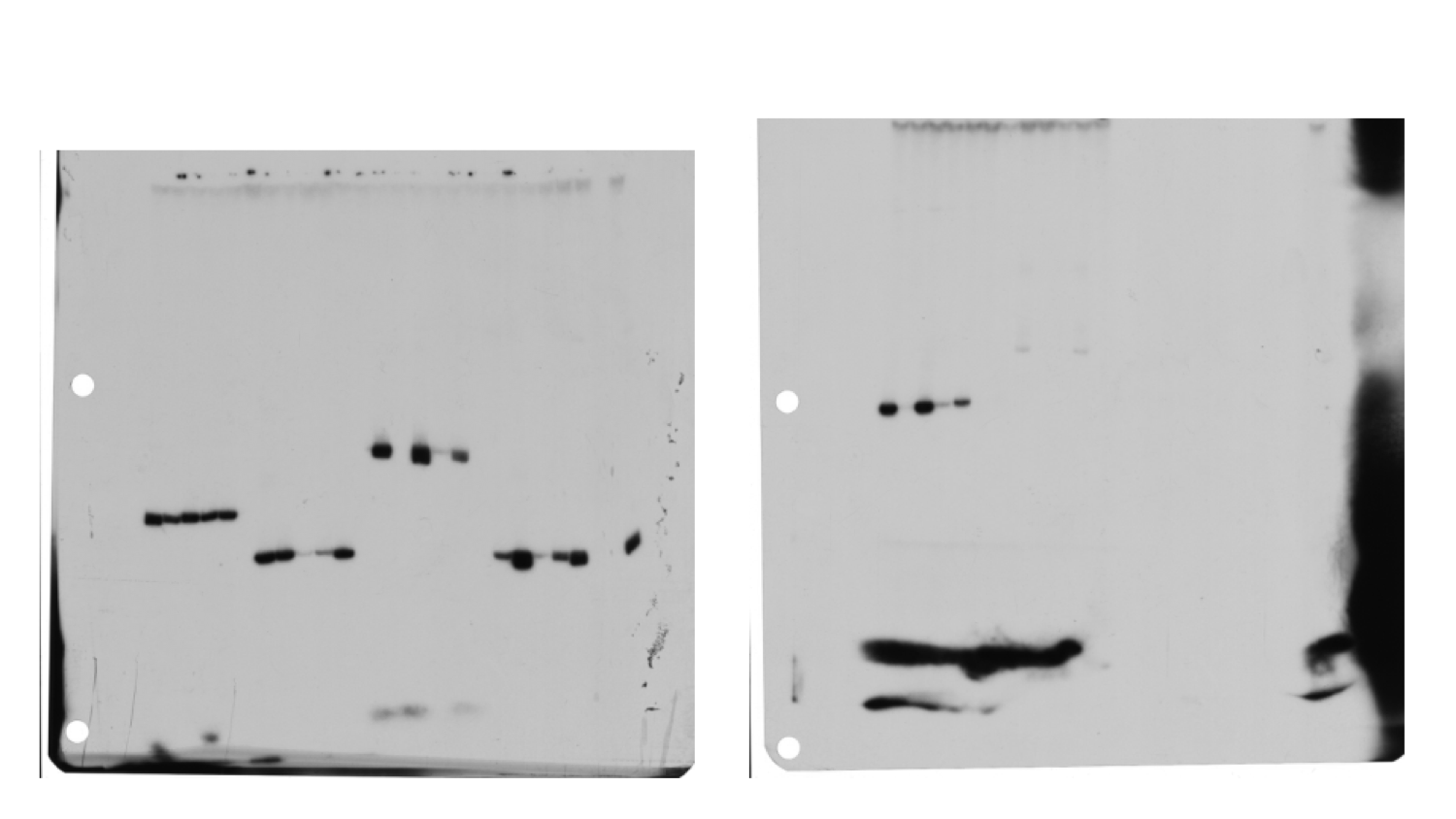

Supplement: Supplementary file 1 — Additional file 1: Figure 1- unprocessed data RT-PCR gels shown in figure 1 [file 12860_2020_282_MOESM1_ESM.tiff]

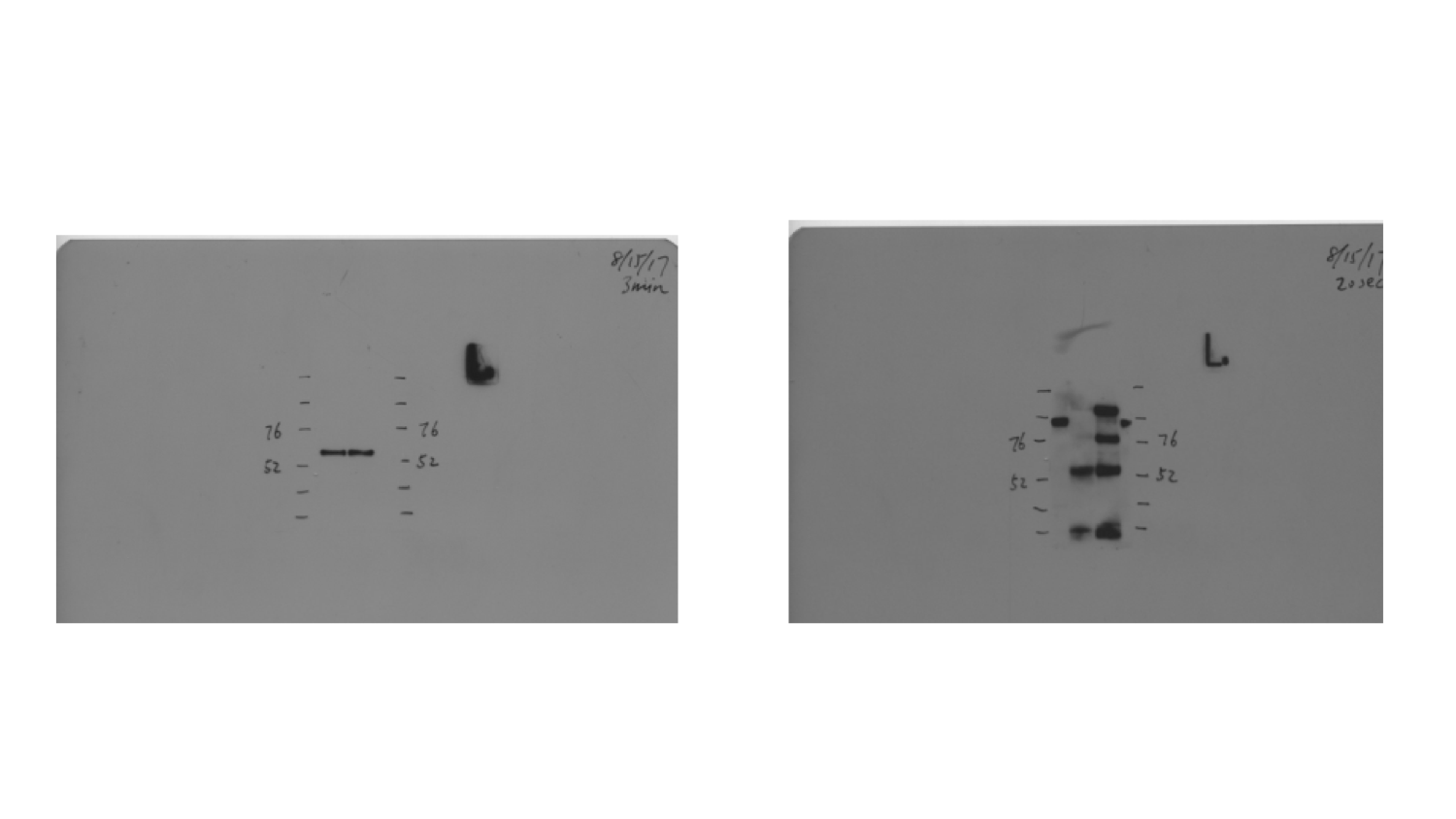

Supplement: Supplementary file 2 — Additional file 2: Figure 2A- unprocessed data. Western blots shown in Figure 2A. Figure 2B- unprocessed data Western blots shown in 2B. Figure 2C- unprocessed data RT-PCR gel shown in Figure 2C. Figure 2D- unprocessed data Western blots shown in Figure 2D. [file 12860_2020_282_MOESM2_ESM.zip › AdditionalFile2A.tiff]

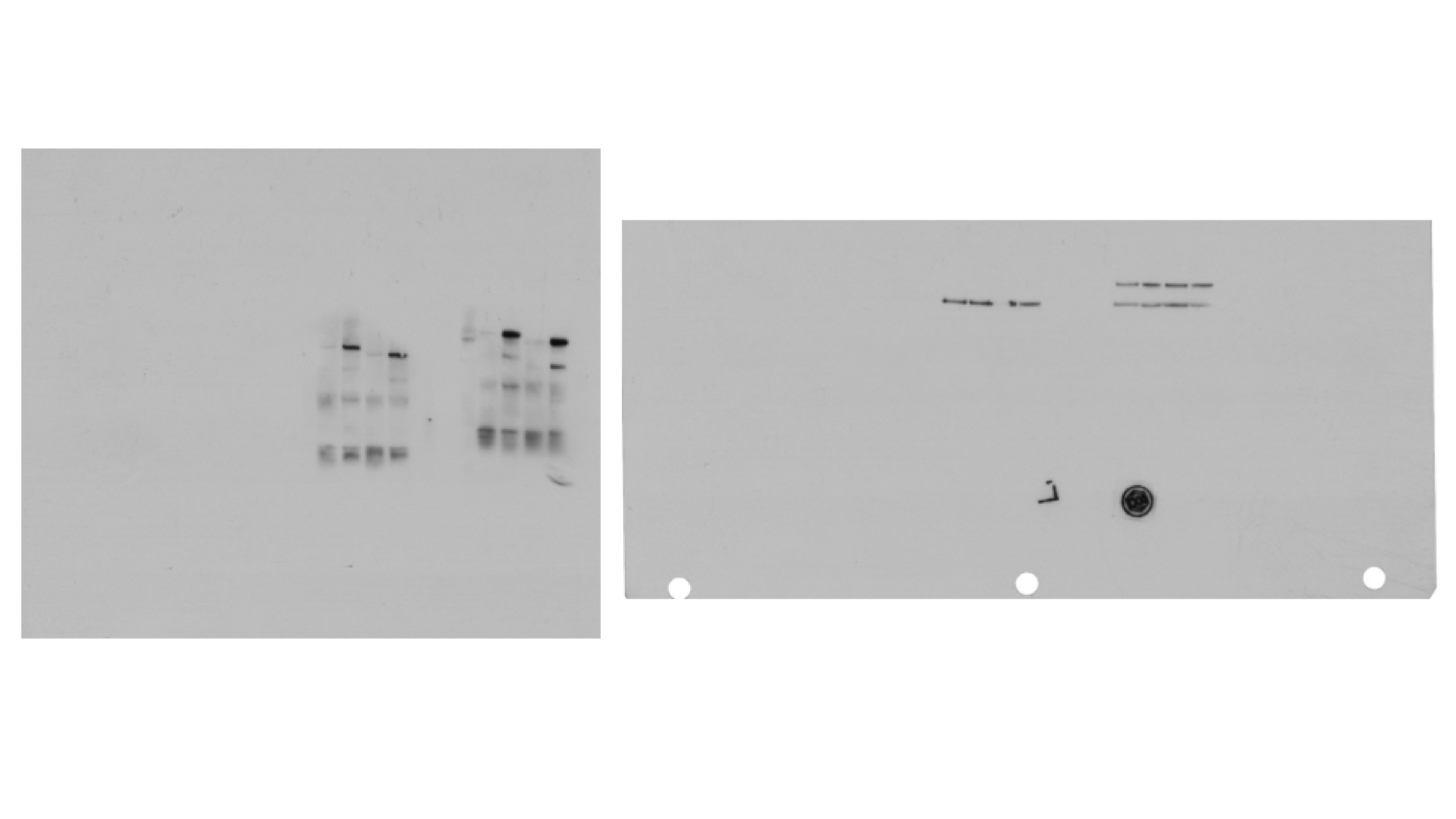

Supplement: Supplementary file 2 — Additional file 2: Figure 2A- unprocessed data. Western blots shown in Figure 2A. Figure 2B- unprocessed data Western blots shown in 2B. Figure 2C- unprocessed data RT-PCR gel shown in Figure 2C. Figure 2D- unprocessed data Western blots shown in Figure 2D. [file 12860_2020_282_MOESM2_ESM.zip › AdditionalFile2B.tiff]

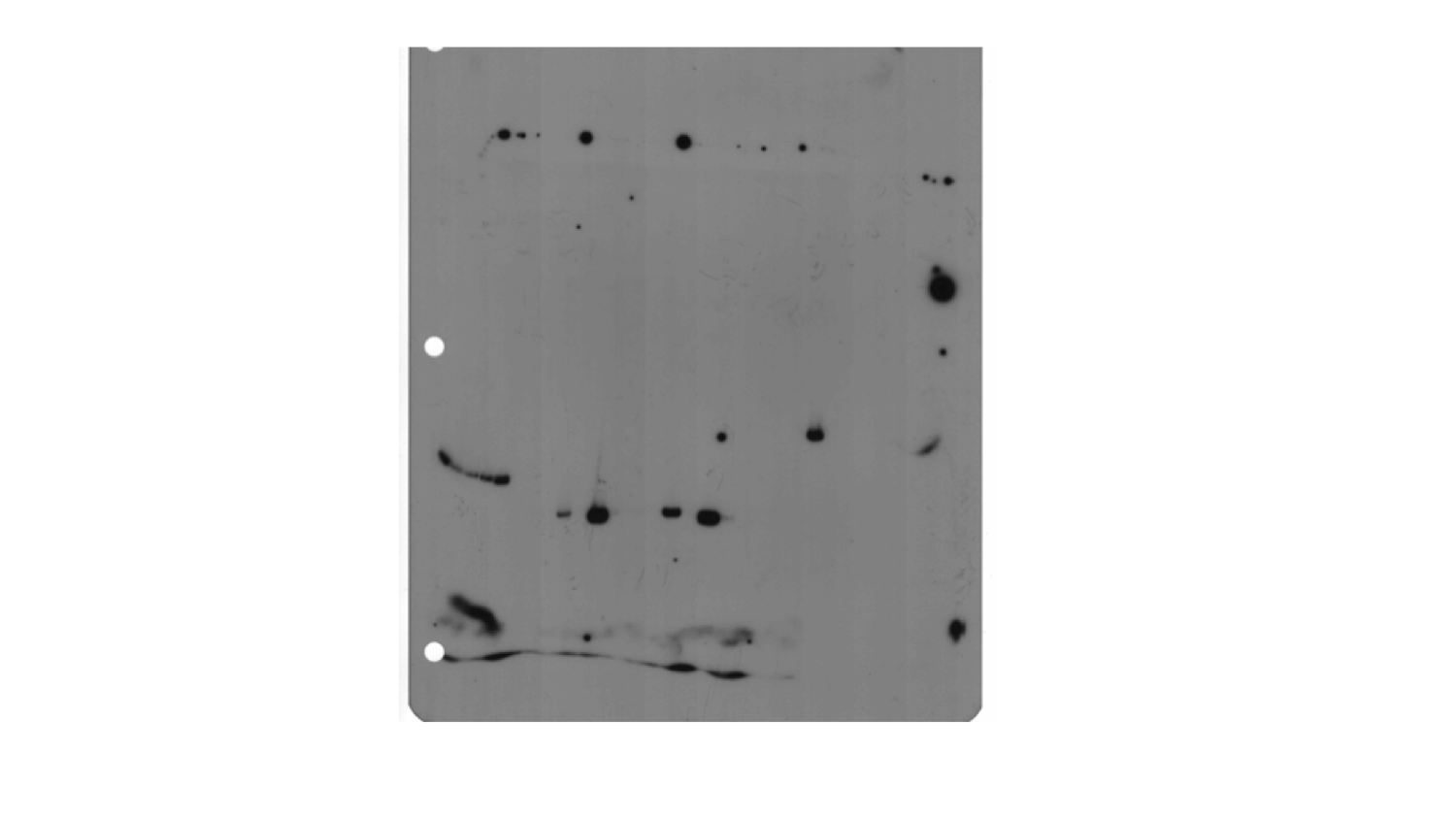

Supplement: Supplementary file 2 — Additional file 2: Figure 2A- unprocessed data. Western blots shown in Figure 2A. Figure 2B- unprocessed data Western blots shown in 2B. Figure 2C- unprocessed data RT-PCR gel shown in Figure 2C. Figure 2D- unprocessed data Western blots shown in Figure 2D. [file 12860_2020_282_MOESM2_ESM.zip › AdditionalFile2C.tiff]

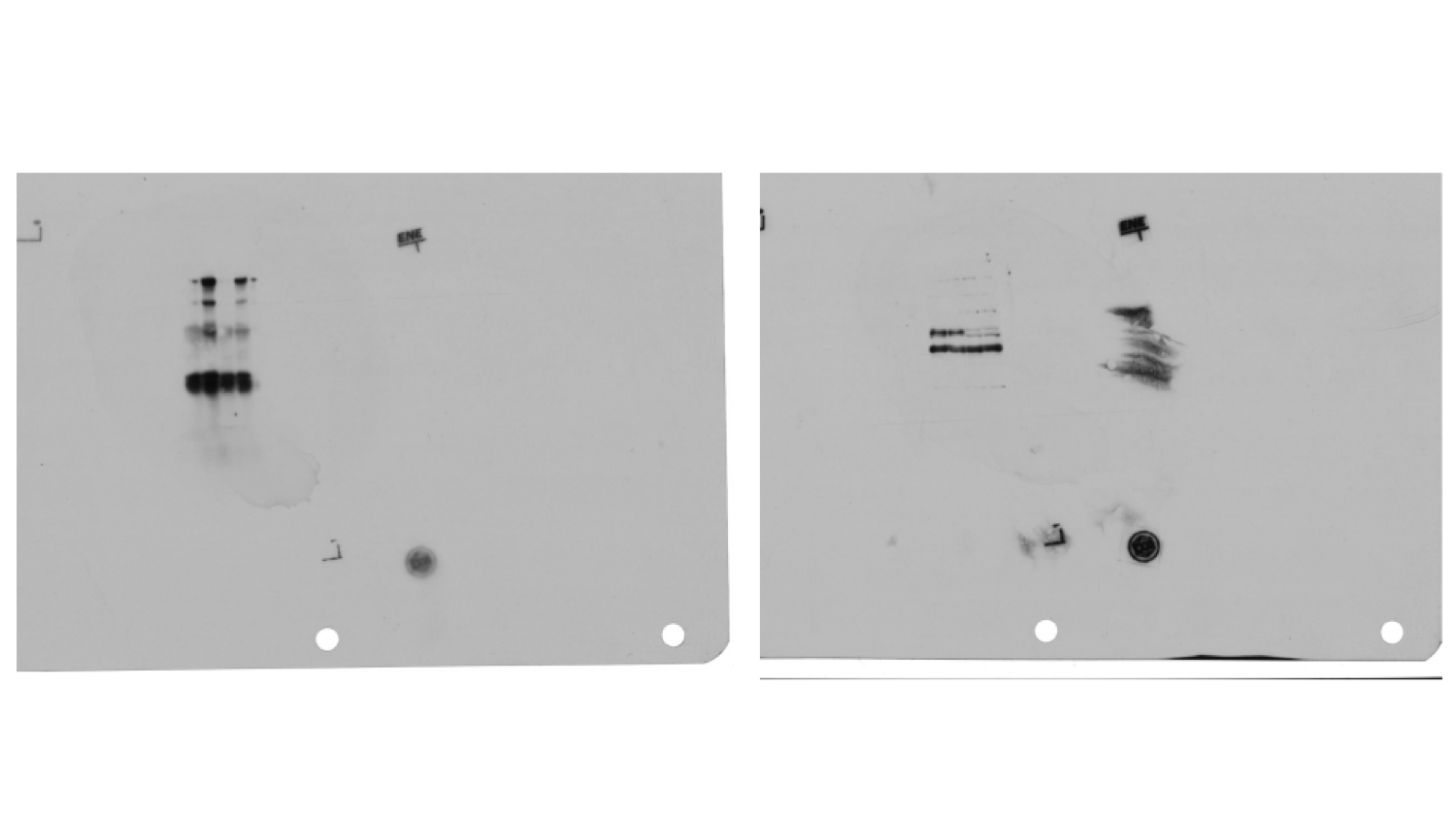

Supplement: Supplementary file 2 — Additional file 2: Figure 2A- unprocessed data. Western blots shown in Figure 2A. Figure 2B- unprocessed data Western blots shown in 2B. Figure 2C- unprocessed data RT-PCR gel shown in Figure 2C. Figure 2D- unprocessed data Western blots shown in Figure 2D. [file 12860_2020_282_MOESM2_ESM.zip › AdditionalFile2D.tiff]

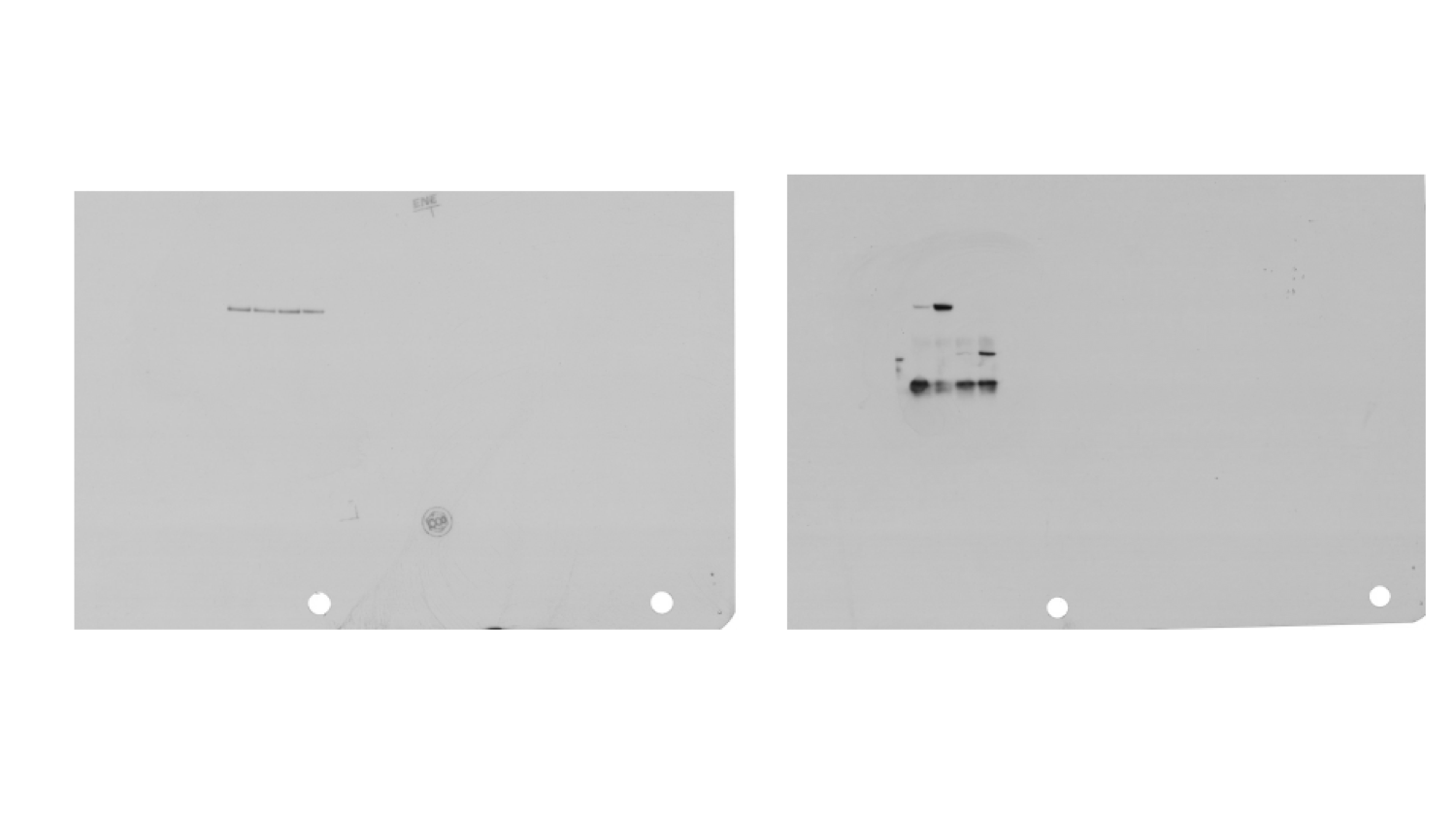

Supplement: Supplementary file 3 — Additional file 3: Figure 4A- unprocessed data. Western blots shown in figure 4A. Figure 4B- unprocessed data Western blots shown in figure 4B. Figure 4C- unprocessed data Western blots shown in figure 4C. Figure 4D- unprocessed data Western blots shown in figure 4D. Figure 4E- unprocessed data Western blots shown in figure 4E. Figure 4F- unprocessed data Western blots shown in figure 4F. Figure 4G- unprocessed data Western blots shown in figure 4G. Figure 4H- unprocessed data Western blots shown in figure 4H. Figure 4I- unprocessed data Western blots shown in figure 4I. Figure 4J- unprocessed data Western blots shown in figure 4J [file 12860_2020_282_MOESM3_ESM.zip › AdditionalFile4A.tiff]

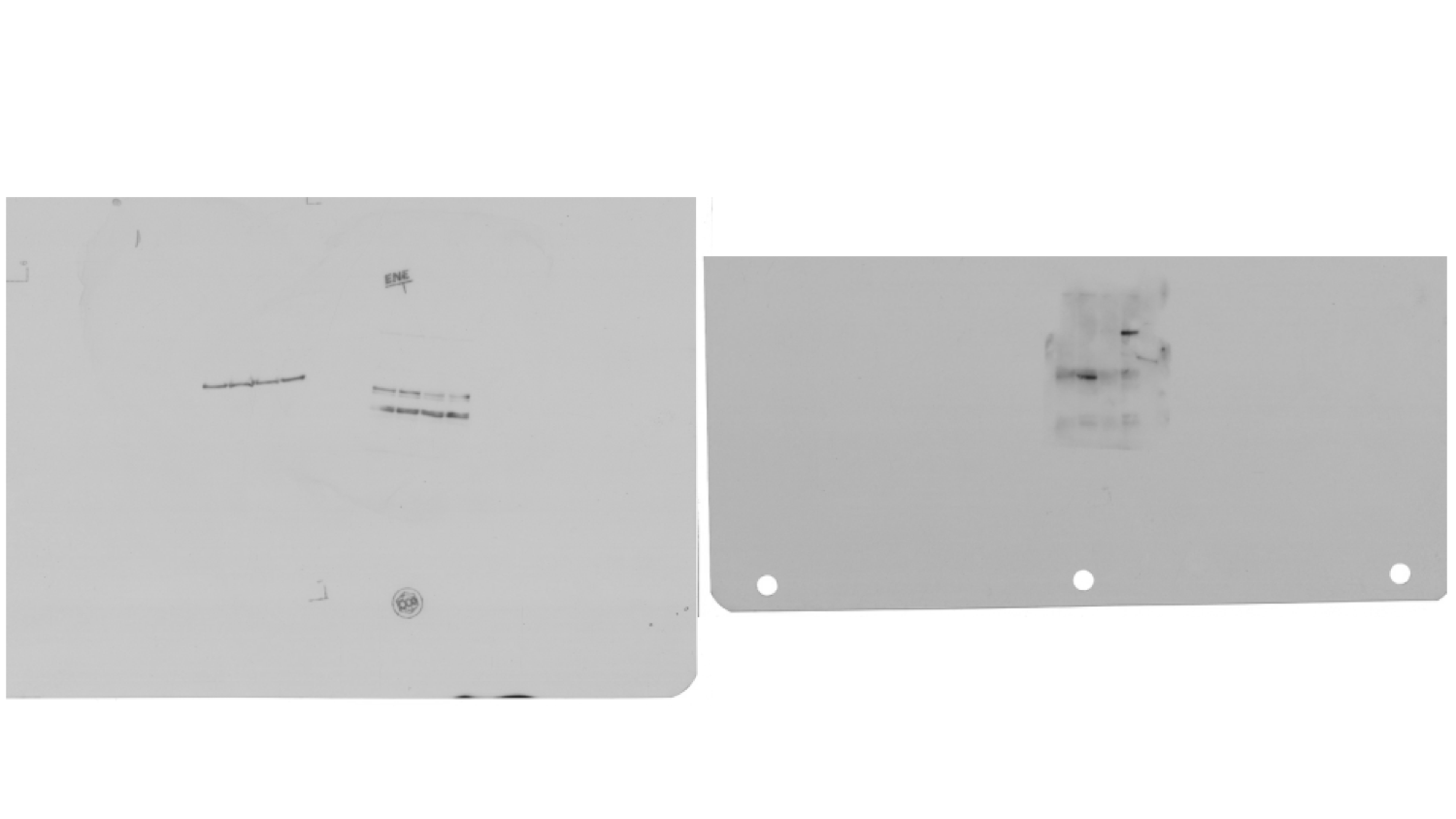

Supplement: Supplementary file 3 — Additional file 3: Figure 4A- unprocessed data. Western blots shown in figure 4A. Figure 4B- unprocessed data Western blots shown in figure 4B. Figure 4C- unprocessed data Western blots shown in figure 4C. Figure 4D- unprocessed data Western blots shown in figure 4D. Figure 4E- unprocessed data Western blots shown in figure 4E. Figure 4F- unprocessed data Western blots shown in figure 4F. Figure 4G- unprocessed data Western blots shown in figure 4G. Figure 4H- unprocessed data Western blots shown in figure 4H. Figure 4I- unprocessed data Western blots shown in figure 4I. Figure 4J- unprocessed data Western blots shown in figure 4J [file 12860_2020_282_MOESM3_ESM.zip › AdditionalFile4B.tiff]

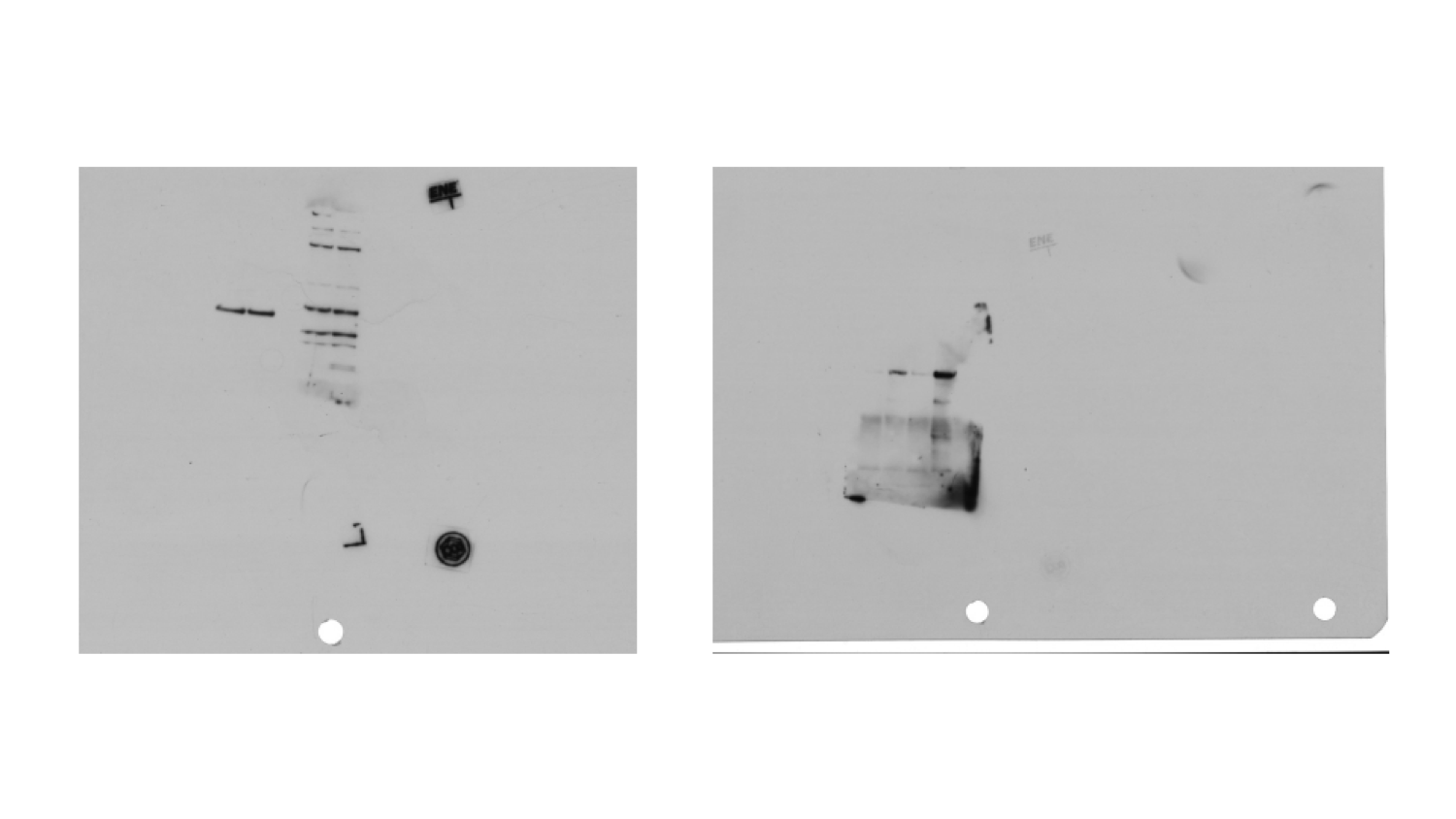

Supplement: Supplementary file 3 — Additional file 3: Figure 4A- unprocessed data. Western blots shown in figure 4A. Figure 4B- unprocessed data Western blots shown in figure 4B. Figure 4C- unprocessed data Western blots shown in figure 4C. Figure 4D- unprocessed data Western blots shown in figure 4D. Figure 4E- unprocessed data Western blots shown in figure 4E. Figure 4F- unprocessed data Western blots shown in figure 4F. Figure 4G- unprocessed data Western blots shown in figure 4G. Figure 4H- unprocessed data Western blots shown in figure 4H. Figure 4I- unprocessed data Western blots shown in figure 4I. Figure 4J- unprocessed data Western blots shown in figure 4J [file 12860_2020_282_MOESM3_ESM.zip › AdditionalFile4C.tiff]

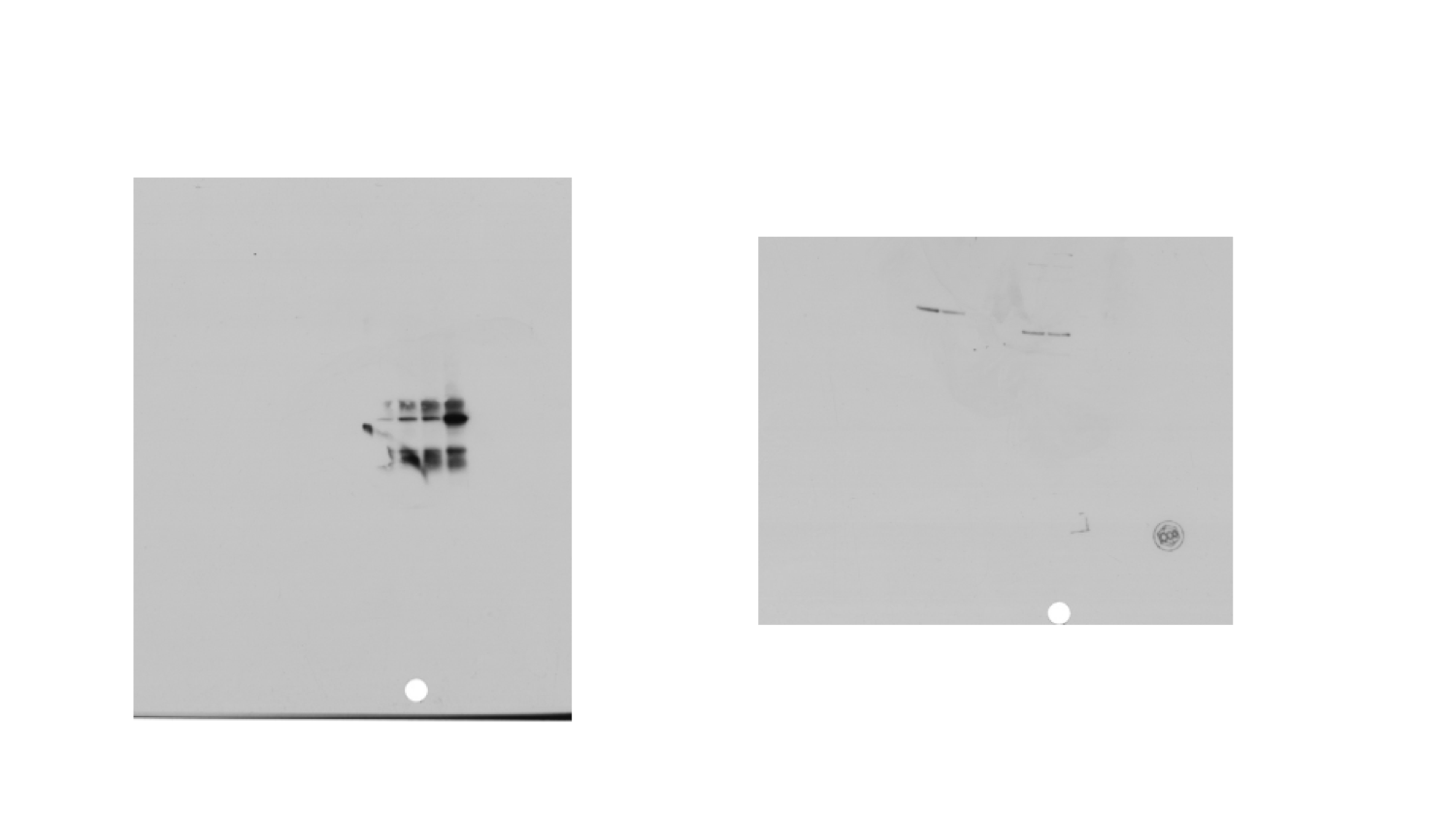

Supplement: Supplementary file 3 — Additional file 3: Figure 4A- unprocessed data. Western blots shown in figure 4A. Figure 4B- unprocessed data Western blots shown in figure 4B. Figure 4C- unprocessed data Western blots shown in figure 4C. Figure 4D- unprocessed data Western blots shown in figure 4D. Figure 4E- unprocessed data Western blots shown in figure 4E. Figure 4F- unprocessed data Western blots shown in figure 4F. Figure 4G- unprocessed data Western blots shown in figure 4G. Figure 4H- unprocessed data Western blots shown in figure 4H. Figure 4I- unprocessed data Western blots shown in figure 4I. Figure 4J- unprocessed data Western blots shown in figure 4J [file 12860_2020_282_MOESM3_ESM.zip › AdditionalFile4D.tiff]

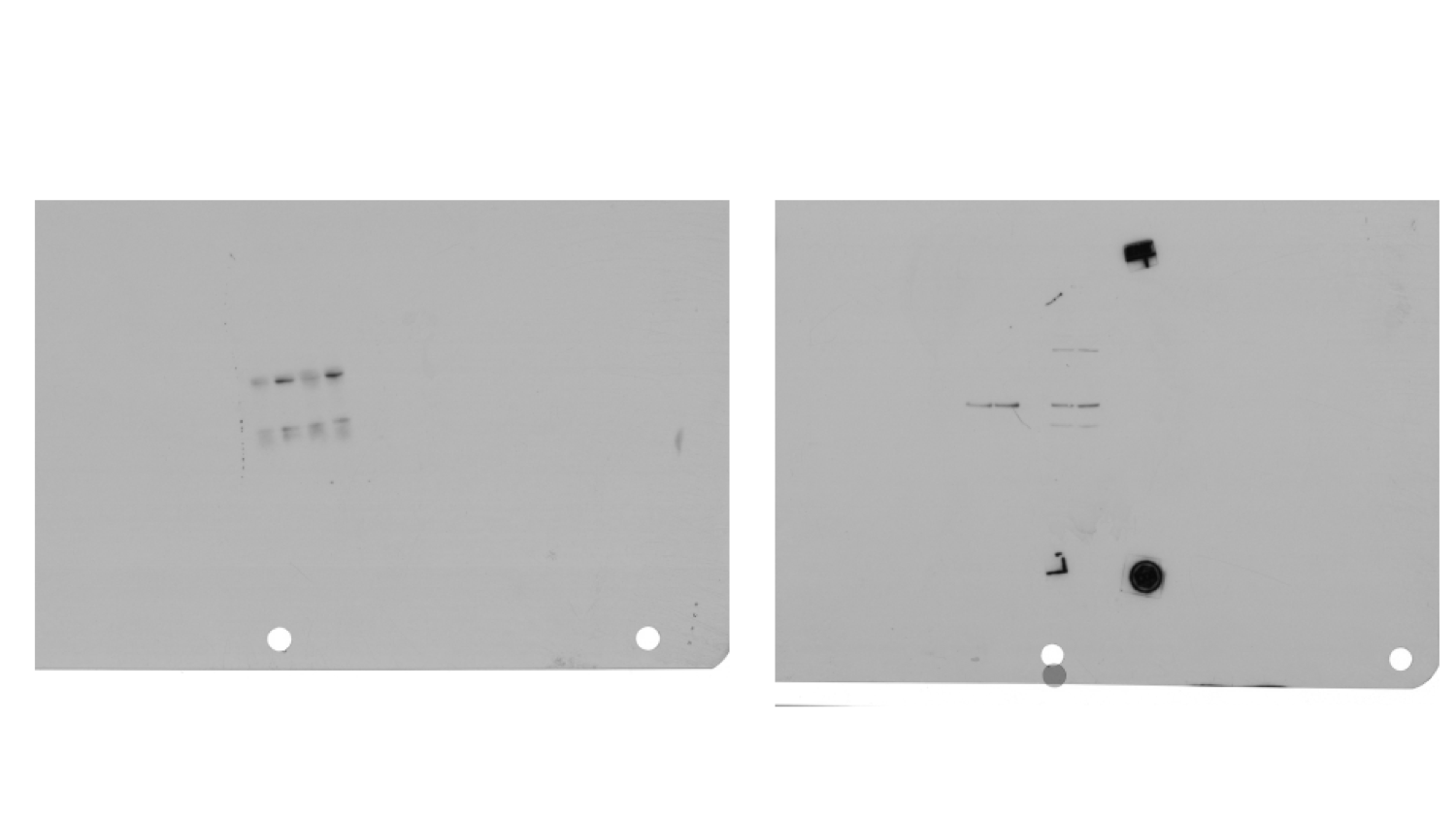

Supplement: Supplementary file 3 — Additional file 3: Figure 4A- unprocessed data. Western blots shown in figure 4A. Figure 4B- unprocessed data Western blots shown in figure 4B. Figure 4C- unprocessed data Western blots shown in figure 4C. Figure 4D- unprocessed data Western blots shown in figure 4D. Figure 4E- unprocessed data Western blots shown in figure 4E. Figure 4F- unprocessed data Western blots shown in figure 4F. Figure 4G- unprocessed data Western blots shown in figure 4G. Figure 4H- unprocessed data Western blots shown in figure 4H. Figure 4I- unprocessed data Western blots shown in figure 4I. Figure 4J- unprocessed data Western blots shown in figure 4J [file 12860_2020_282_MOESM3_ESM.zip › AdditionalFile4E.tiff]

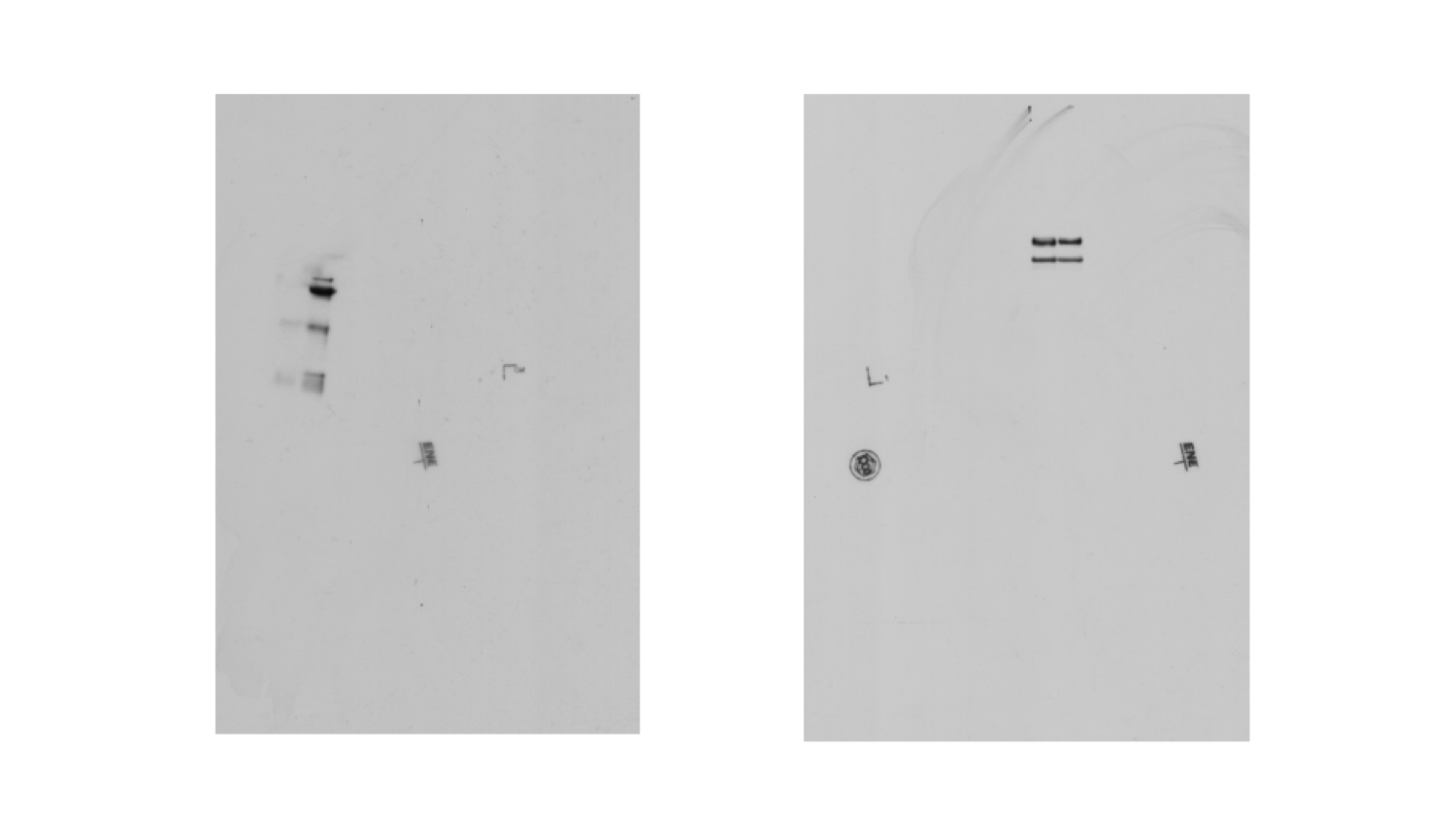

Supplement: Supplementary file 3 — Additional file 3: Figure 4A- unprocessed data. Western blots shown in figure 4A. Figure 4B- unprocessed data Western blots shown in figure 4B. Figure 4C- unprocessed data Western blots shown in figure 4C. Figure 4D- unprocessed data Western blots shown in figure 4D. Figure 4E- unprocessed data Western blots shown in figure 4E. Figure 4F- unprocessed data Western blots shown in figure 4F. Figure 4G- unprocessed data Western blots shown in figure 4G. Figure 4H- unprocessed data Western blots shown in figure 4H. Figure 4I- unprocessed data Western blots shown in figure 4I. Figure 4J- unprocessed data Western blots shown in figure 4J [file 12860_2020_282_MOESM3_ESM.zip › AdditionalFile4F.tiff]

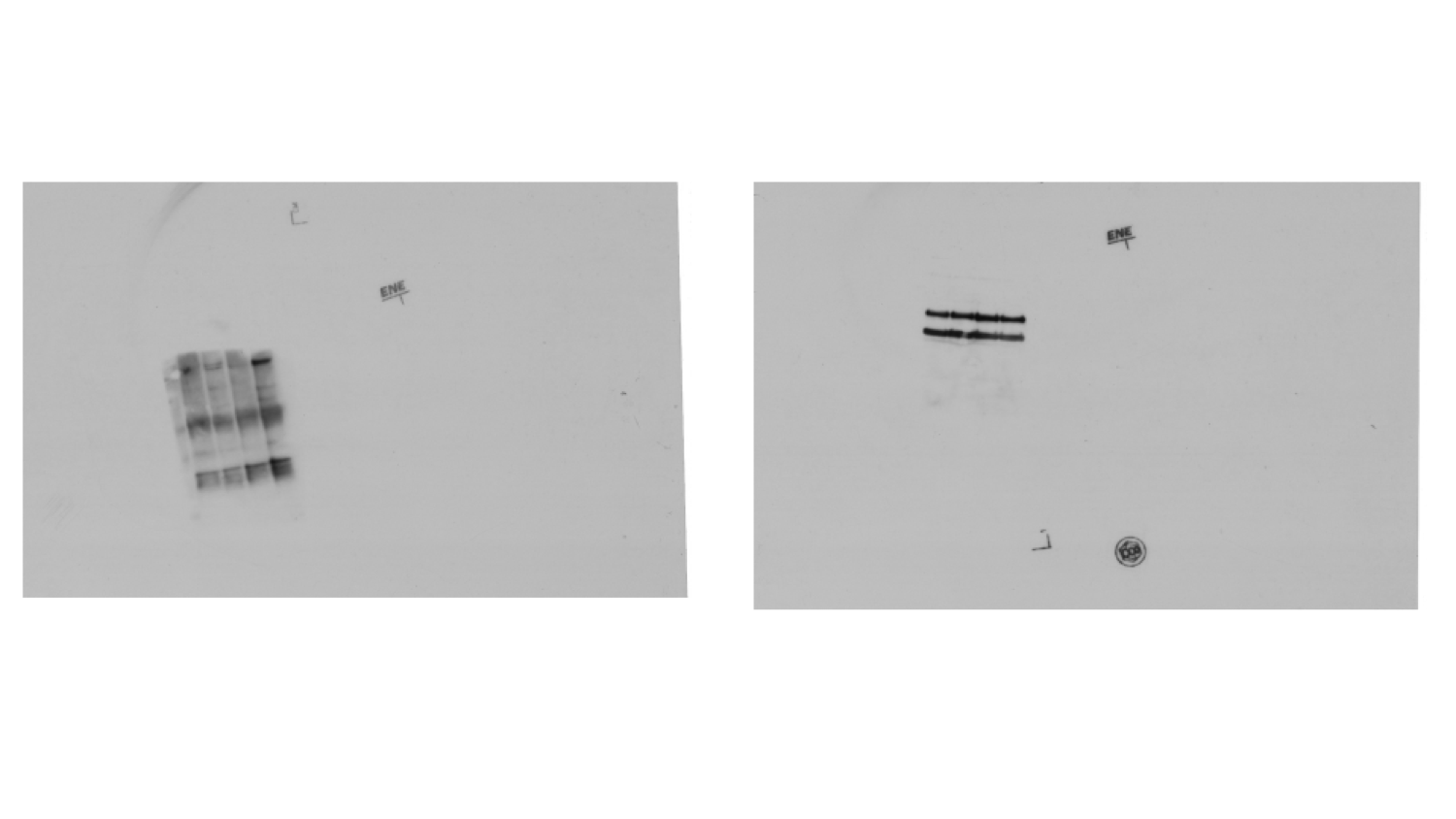

Supplement: Supplementary file 3 — Additional file 3: Figure 4A- unprocessed data. Western blots shown in figure 4A. Figure 4B- unprocessed data Western blots shown in figure 4B. Figure 4C- unprocessed data Western blots shown in figure 4C. Figure 4D- unprocessed data Western blots shown in figure 4D. Figure 4E- unprocessed data Western blots shown in figure 4E. Figure 4F- unprocessed data Western blots shown in figure 4F. Figure 4G- unprocessed data Western blots shown in figure 4G. Figure 4H- unprocessed data Western blots shown in figure 4H. Figure 4I- unprocessed data Western blots shown in figure 4I. Figure 4J- unprocessed data Western blots shown in figure 4J [file 12860_2020_282_MOESM3_ESM.zip › AdditionalFile4G.tiff]

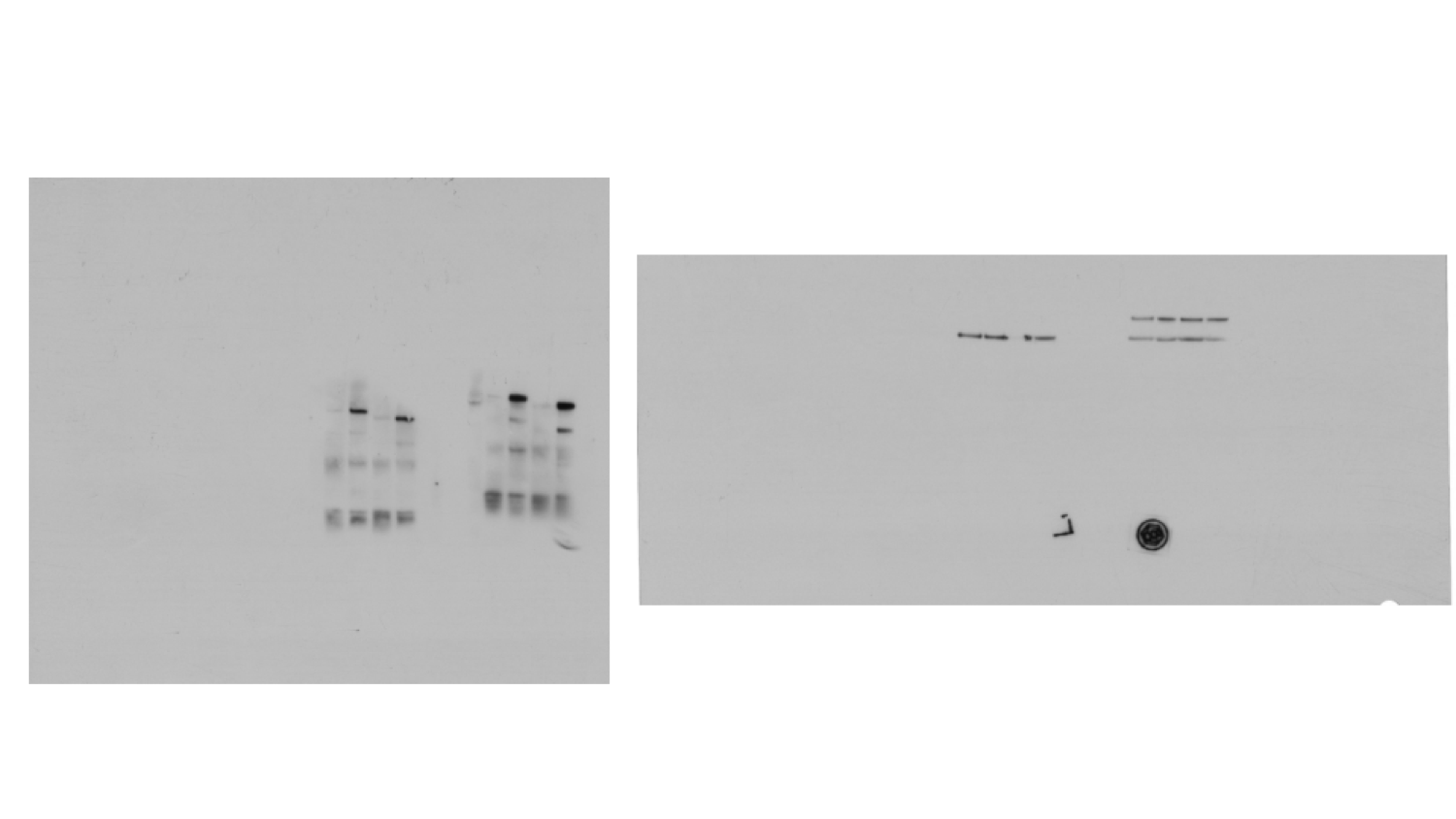

Supplement: Supplementary file 3 — Additional file 3: Figure 4A- unprocessed data. Western blots shown in figure 4A. Figure 4B- unprocessed data Western blots shown in figure 4B. Figure 4C- unprocessed data Western blots shown in figure 4C. Figure 4D- unprocessed data Western blots shown in figure 4D. Figure 4E- unprocessed data Western blots shown in figure 4E. Figure 4F- unprocessed data Western blots shown in figure 4F. Figure 4G- unprocessed data Western blots shown in figure 4G. Figure 4H- unprocessed data Western blots shown in figure 4H. Figure 4I- unprocessed data Western blots shown in figure 4I. Figure 4J- unprocessed data Western blots shown in figure 4J [file 12860_2020_282_MOESM3_ESM.zip › AdditionalFile4H.tiff]

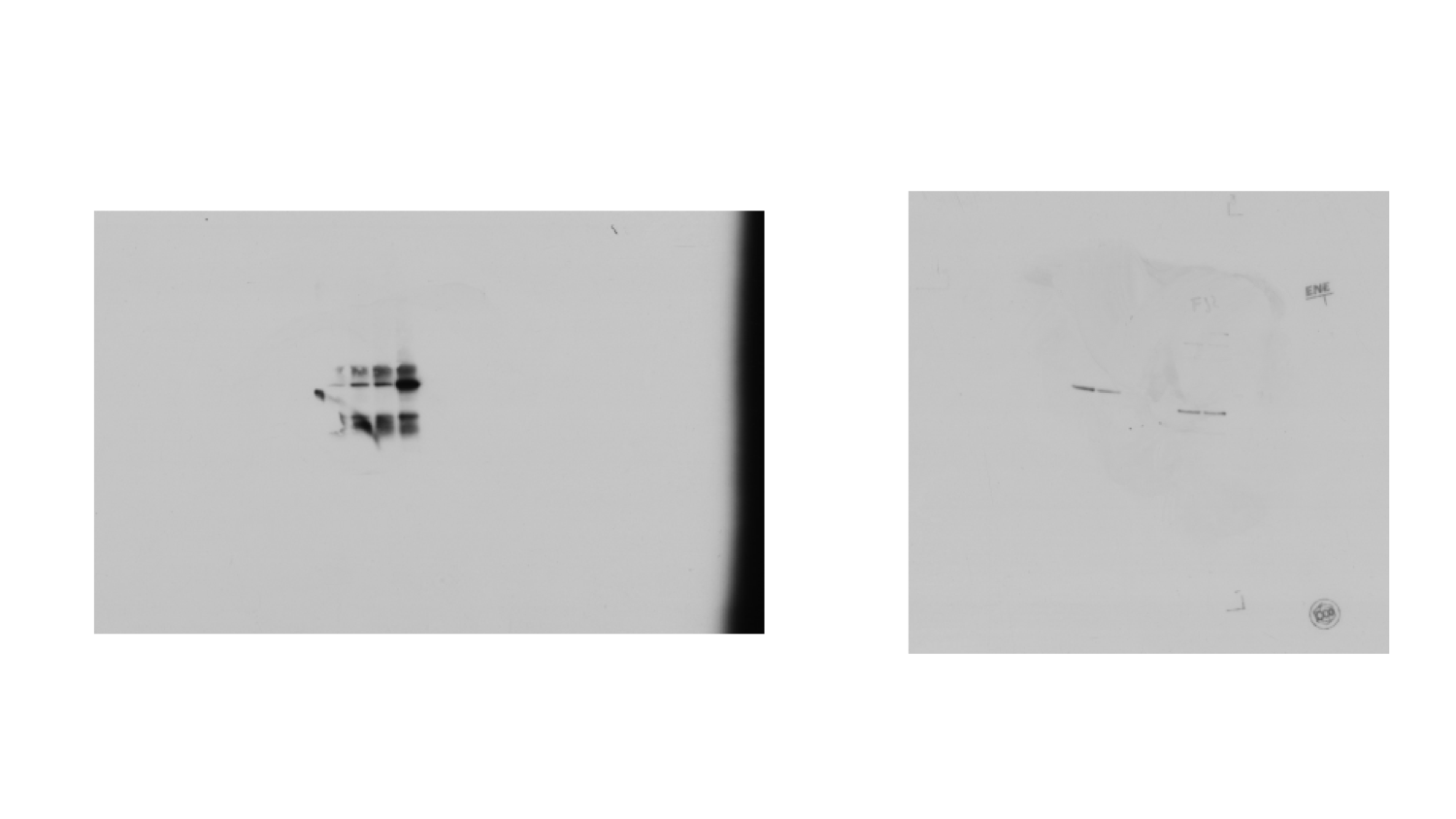

Supplement: Supplementary file 3 — Additional file 3: Figure 4A- unprocessed data. Western blots shown in figure 4A. Figure 4B- unprocessed data Western blots shown in figure 4B. Figure 4C- unprocessed data Western blots shown in figure 4C. Figure 4D- unprocessed data Western blots shown in figure 4D. Figure 4E- unprocessed data Western blots shown in figure 4E. Figure 4F- unprocessed data Western blots shown in figure 4F. Figure 4G- unprocessed data Western blots shown in figure 4G. Figure 4H- unprocessed data Western blots shown in figure 4H. Figure 4I- unprocessed data Western blots shown in figure 4I. Figure 4J- unprocessed data Western blots shown in figure 4J [file 12860_2020_282_MOESM3_ESM.zip › AdditionalFile4I.tiff]

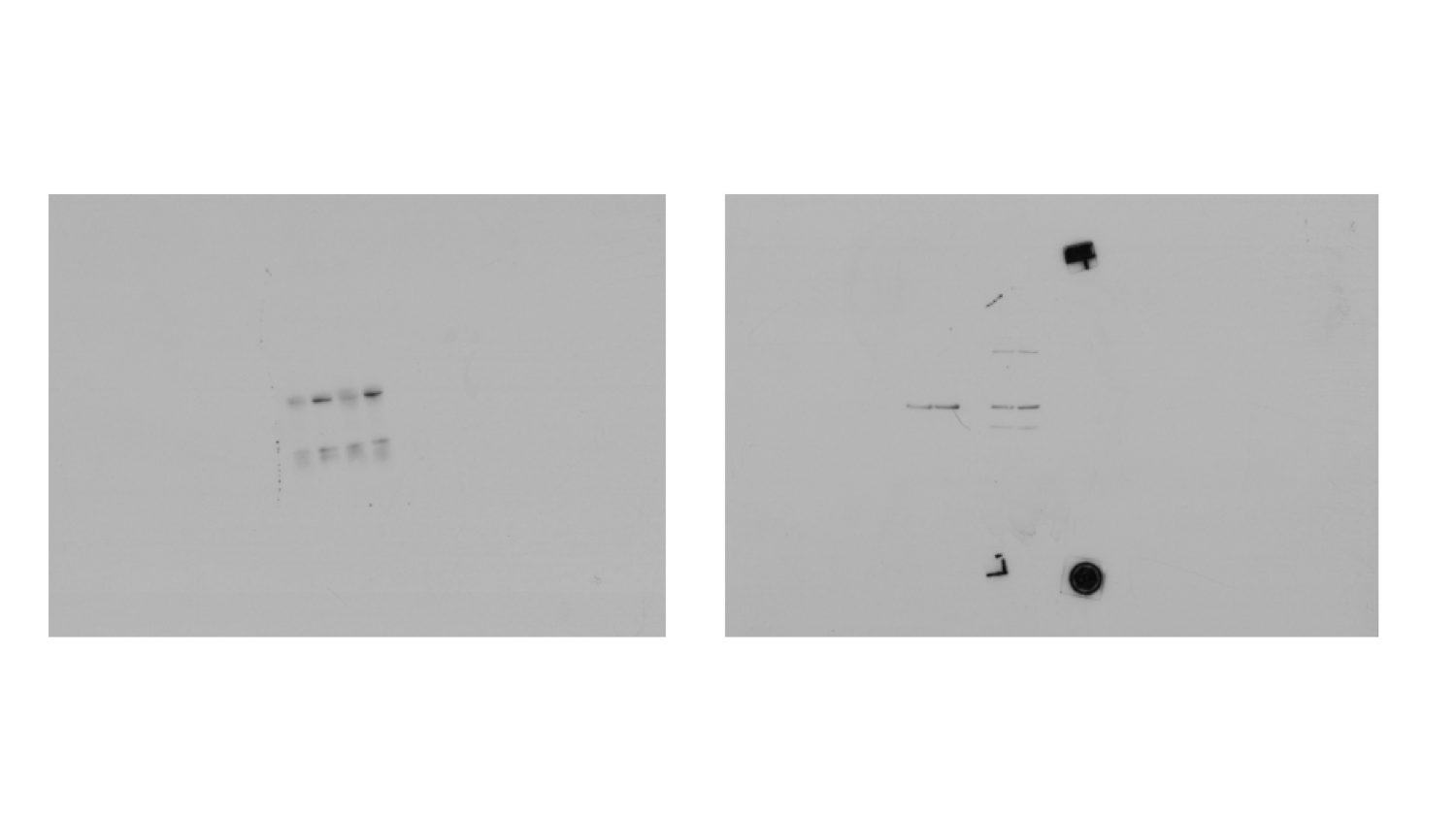

Supplement: Supplementary file 3 — Additional file 3: Figure 4A- unprocessed data. Western blots shown in figure 4A. Figure 4B- unprocessed data Western blots shown in figure 4B. Figure 4C- unprocessed data Western blots shown in figure 4C. Figure 4D- unprocessed data Western blots shown in figure 4D. Figure 4E- unprocessed data Western blots shown in figure 4E. Figure 4F- unprocessed data Western blots shown in figure 4F. Figure 4G- unprocessed data Western blots shown in figure 4G. Figure 4H- unprocessed data Western blots shown in figure 4H. Figure 4I- unprocessed data Western blots shown in figure 4I. Figure 4J- unprocessed data Western blots shown in figure 4J [file 12860_2020_282_MOESM3_ESM.zip › AdditionalFile4J.tiff]

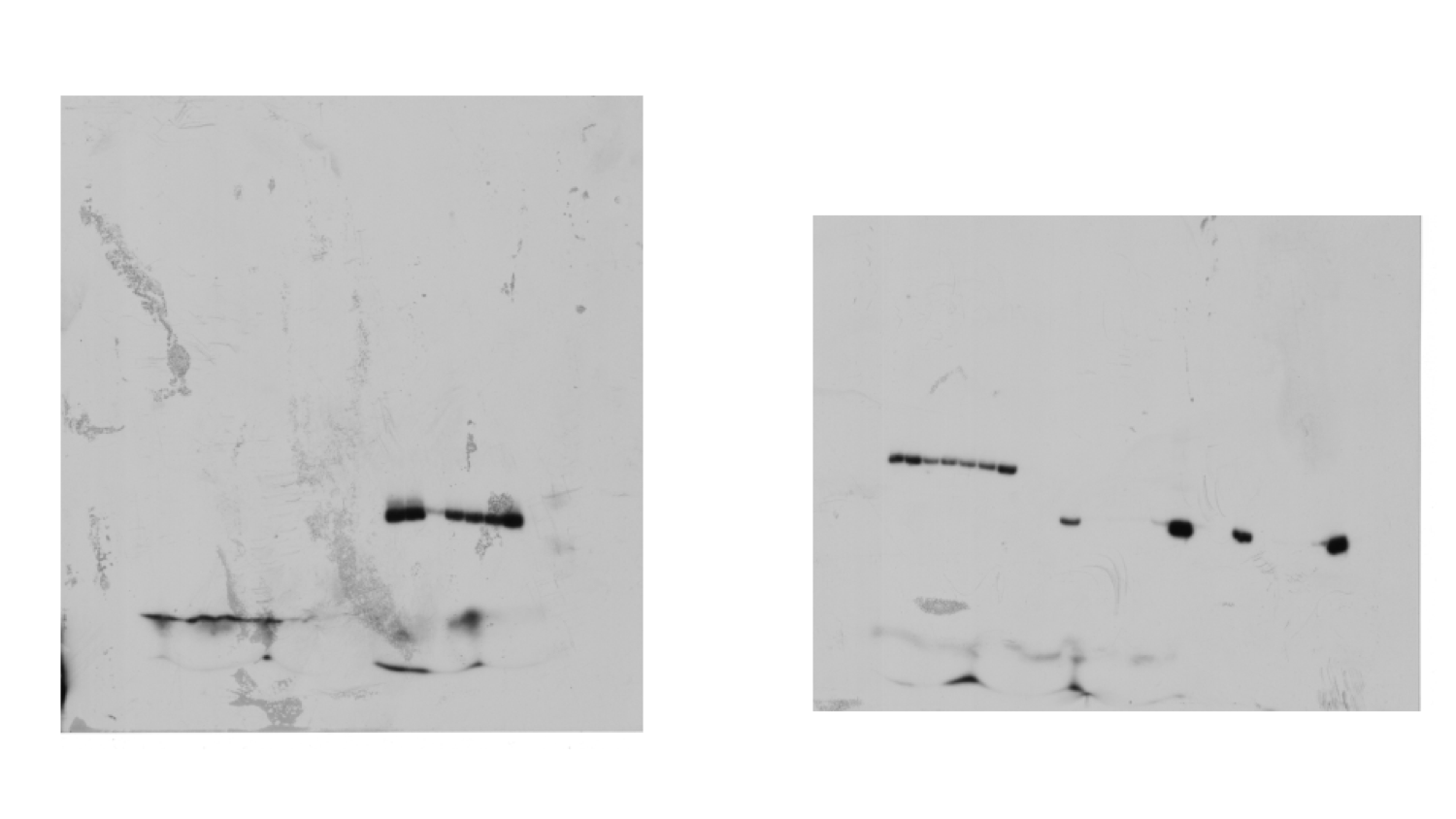

Supplement: Supplementary file 4 — Additional file 4: Figure 5A- unprocessed data. RT-PCR gels shown in figure 5A. Figure 5B- unprocessed data RT-PCR gels shown in figure 5B. Figure 5C- unprocessed data. RT-PCR gels shown in figure 5C. Figure 5D- unprocessed data. RT-PCR gels shown in figure 5D. Figure 5E- unprocessed data. RT-PCR gels shown in figure 5E [file 12860_2020_282_MOESM4_ESM.zip › AdditionalFile5A.tiff]

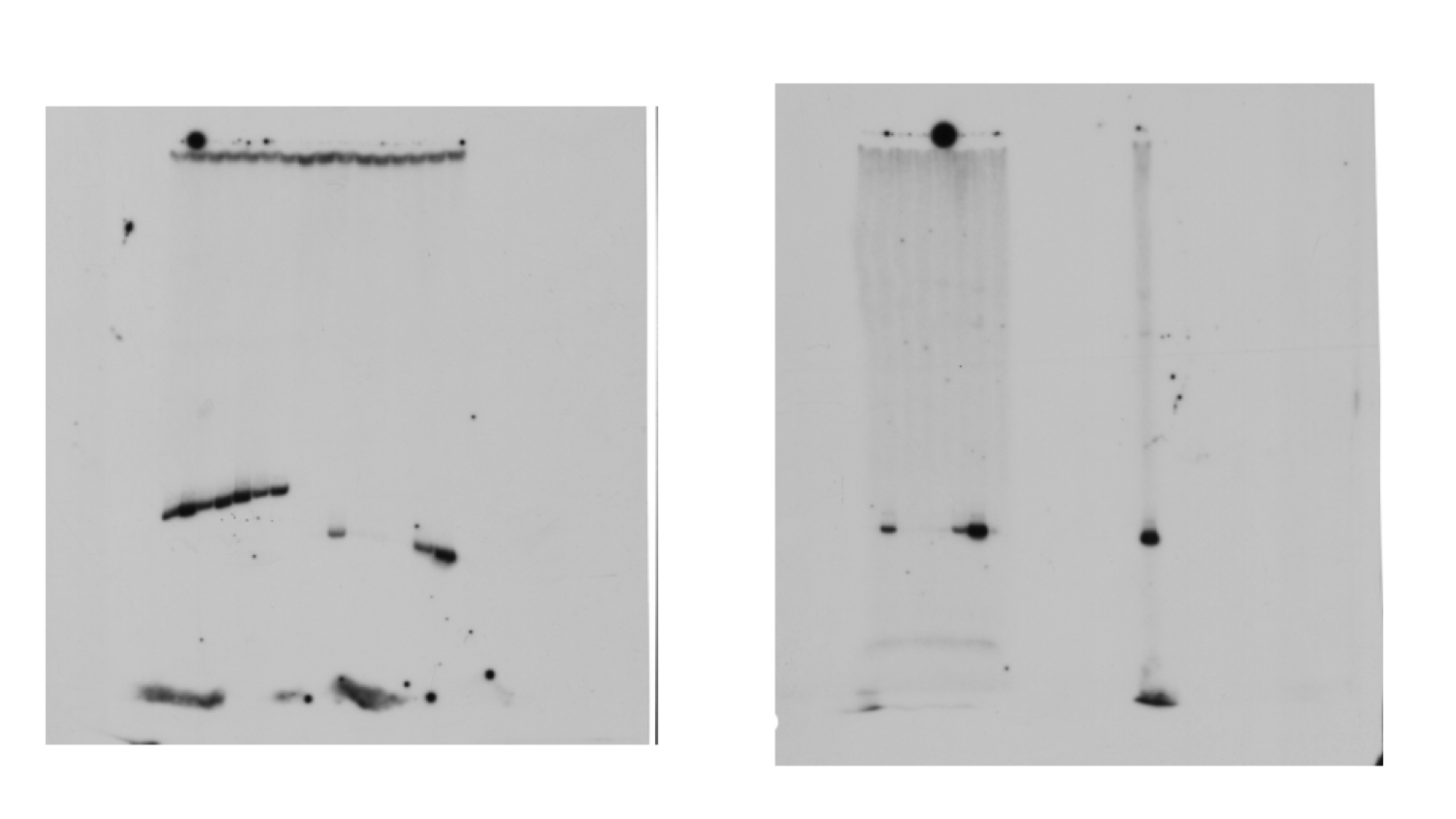

Supplement: Supplementary file 4 — Additional file 4: Figure 5A- unprocessed data. RT-PCR gels shown in figure 5A. Figure 5B- unprocessed data RT-PCR gels shown in figure 5B. Figure 5C- unprocessed data. RT-PCR gels shown in figure 5C. Figure 5D- unprocessed data. RT-PCR gels shown in figure 5D. Figure 5E- unprocessed data. RT-PCR gels shown in figure 5E [file 12860_2020_282_MOESM4_ESM.zip › AdditionalFile5B.tiff]

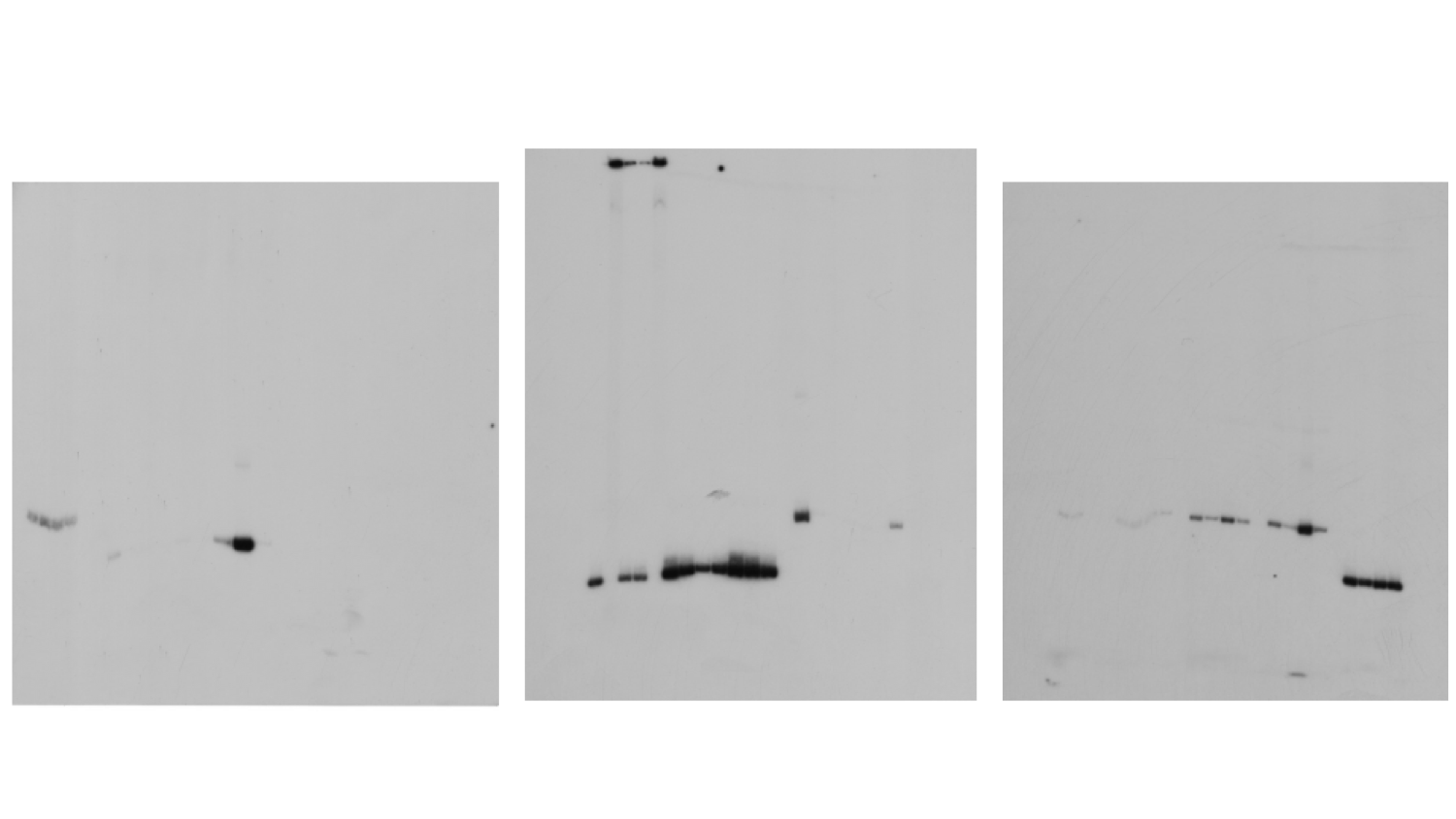

Supplement: Supplementary file 4 — Additional file 4: Figure 5A- unprocessed data. RT-PCR gels shown in figure 5A. Figure 5B- unprocessed data RT-PCR gels shown in figure 5B. Figure 5C- unprocessed data. RT-PCR gels shown in figure 5C. Figure 5D- unprocessed data. RT-PCR gels shown in figure 5D. Figure 5E- unprocessed data. RT-PCR gels shown in figure 5E [file 12860_2020_282_MOESM4_ESM.zip › AdditionalFile5C.tiff]

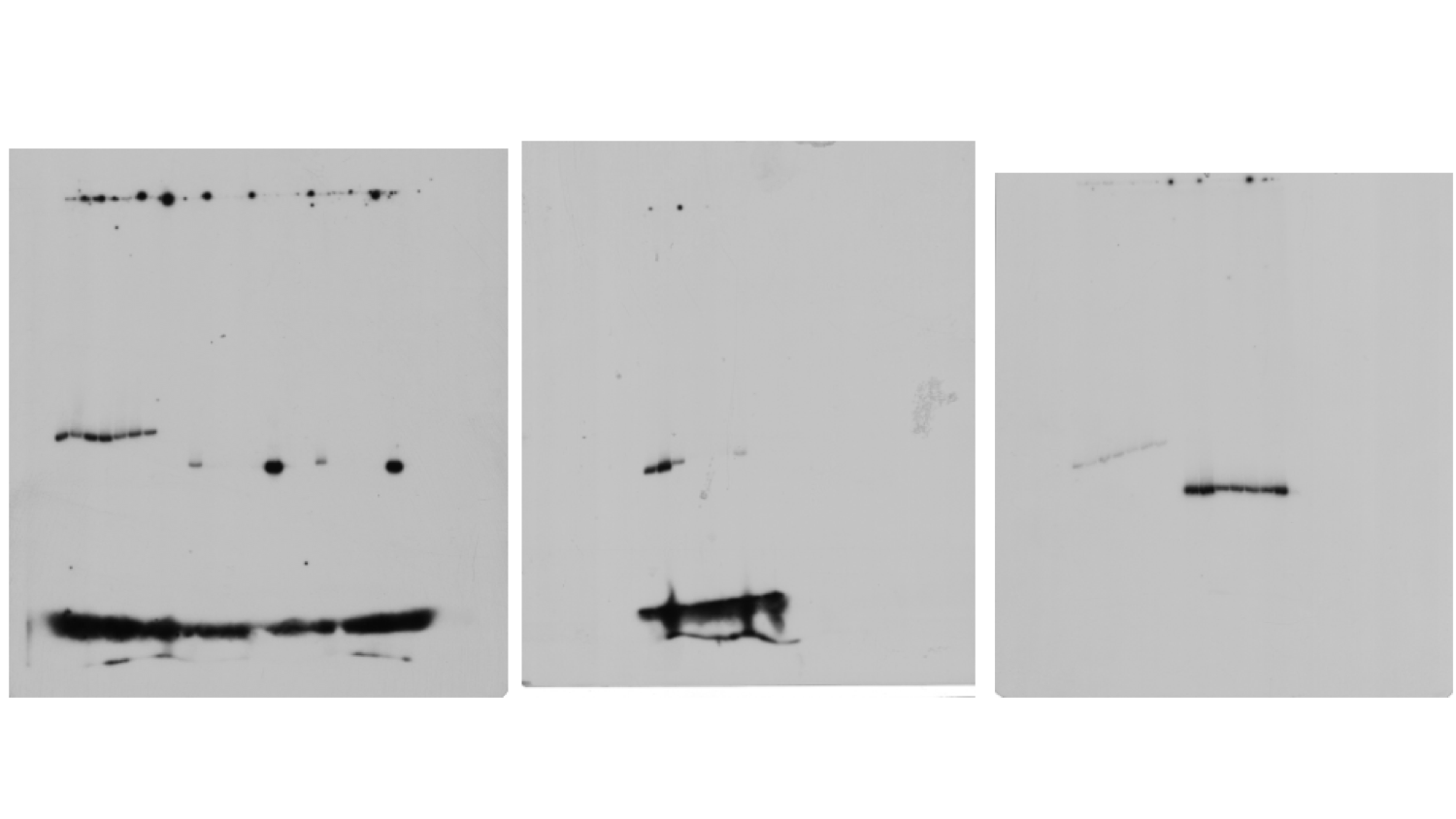

Supplement: Supplementary file 4 — Additional file 4: Figure 5A- unprocessed data. RT-PCR gels shown in figure 5A. Figure 5B- unprocessed data RT-PCR gels shown in figure 5B. Figure 5C- unprocessed data. RT-PCR gels shown in figure 5C. Figure 5D- unprocessed data. RT-PCR gels shown in figure 5D. Figure 5E- unprocessed data. RT-PCR gels shown in figure 5E [file 12860_2020_282_MOESM4_ESM.zip › AdditionalFile5D.tiff]

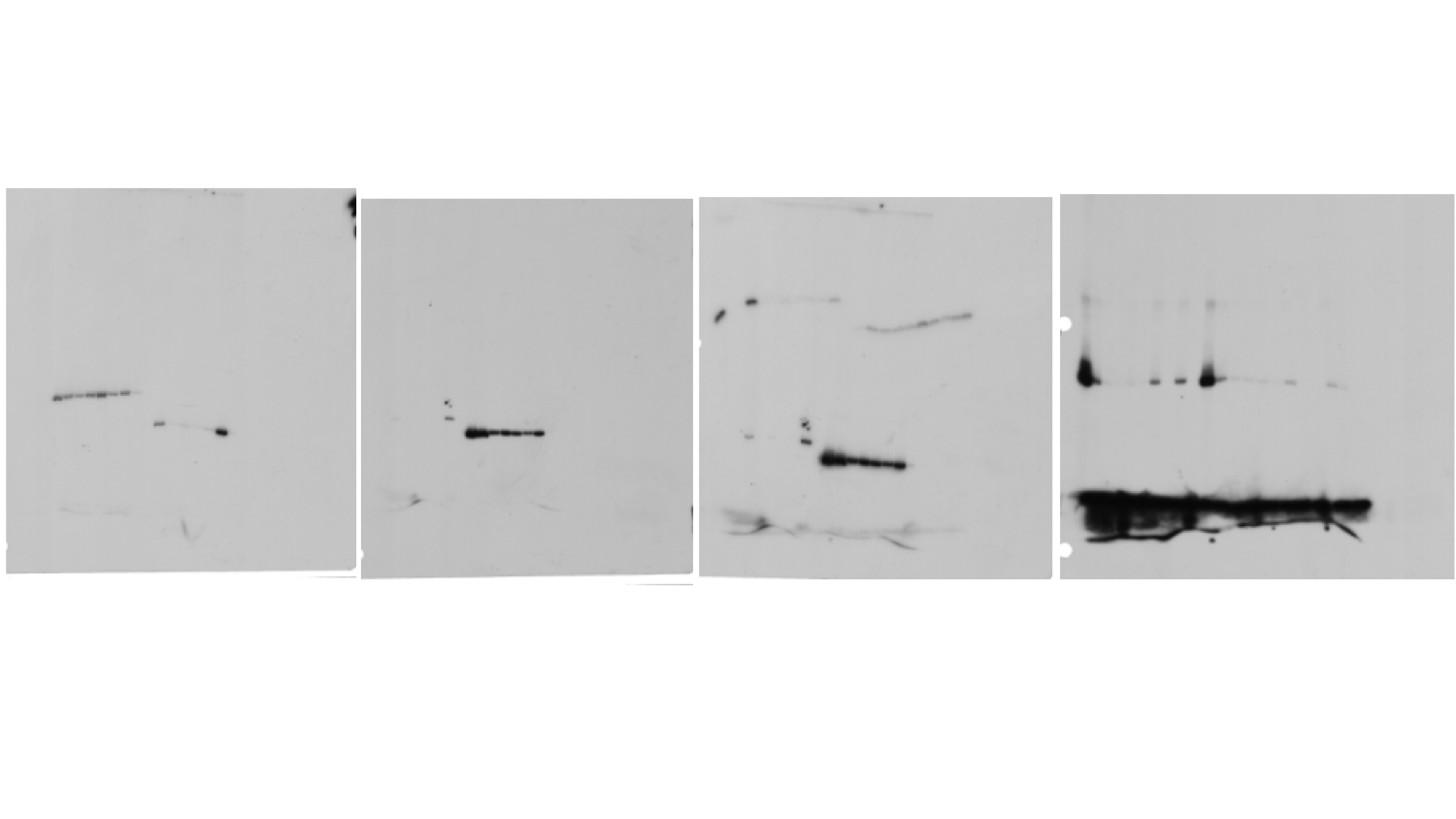

Supplement: Supplementary file 4 — Additional file 4: Figure 5A- unprocessed data. RT-PCR gels shown in figure 5A. Figure 5B- unprocessed data RT-PCR gels shown in figure 5B. Figure 5C- unprocessed data. RT-PCR gels shown in figure 5C. Figure 5D- unprocessed data. RT-PCR gels shown in figure 5D. Figure 5E- unprocessed data. RT-PCR gels shown in figure 5E [file 12860_2020_282_MOESM4_ESM.zip › AdditionalFile5E.tiff]

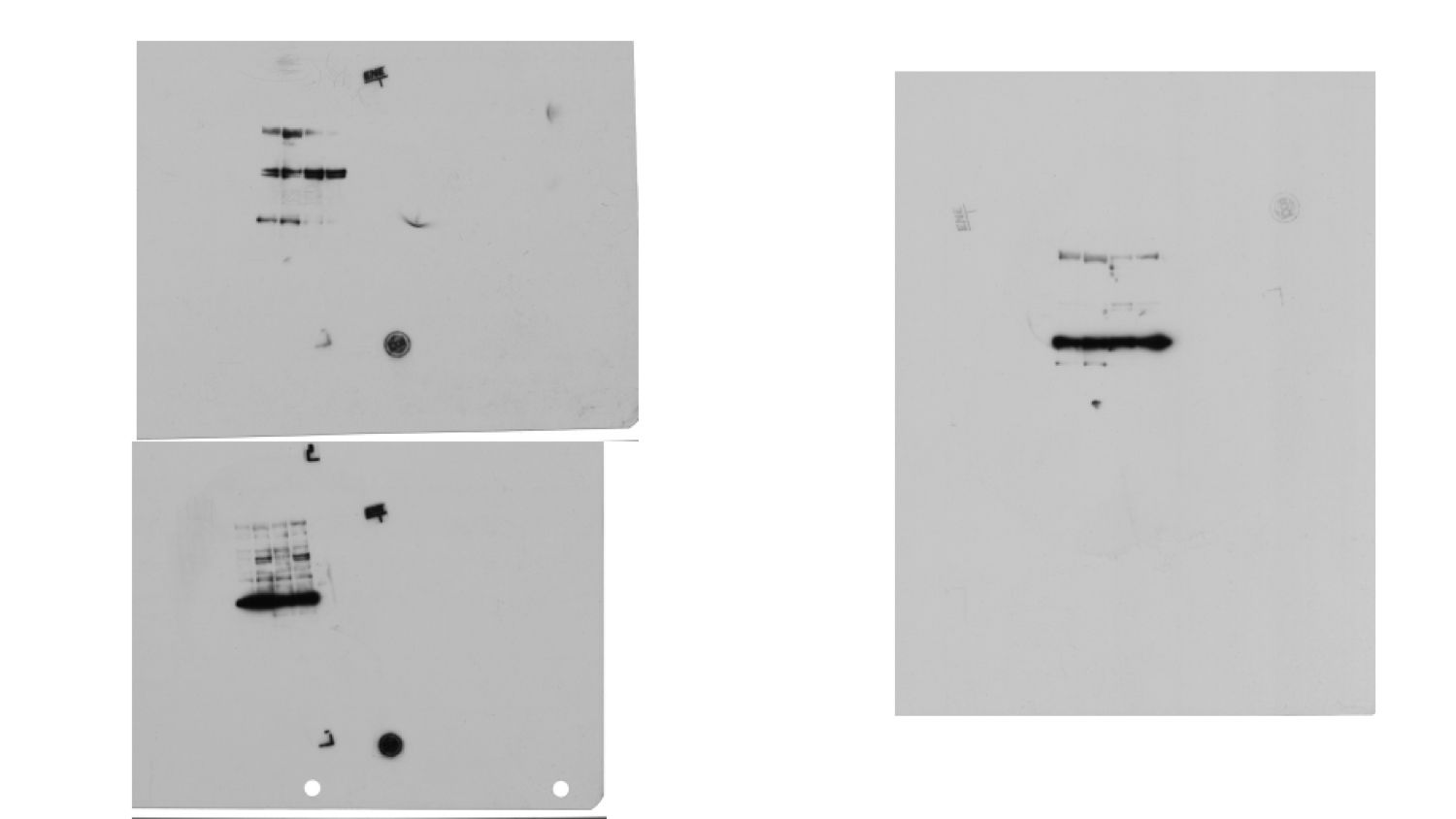

Supplement: Supplementary file 5 — Additional file 5: Figure 6A- unprocessed data. Western blots shown in figure 6A. Figure 6B- unprocessed data Western blots shown in figure 6B [file 12860_2020_282_MOESM5_ESM.zip › AdditionalFile6A.tiff]

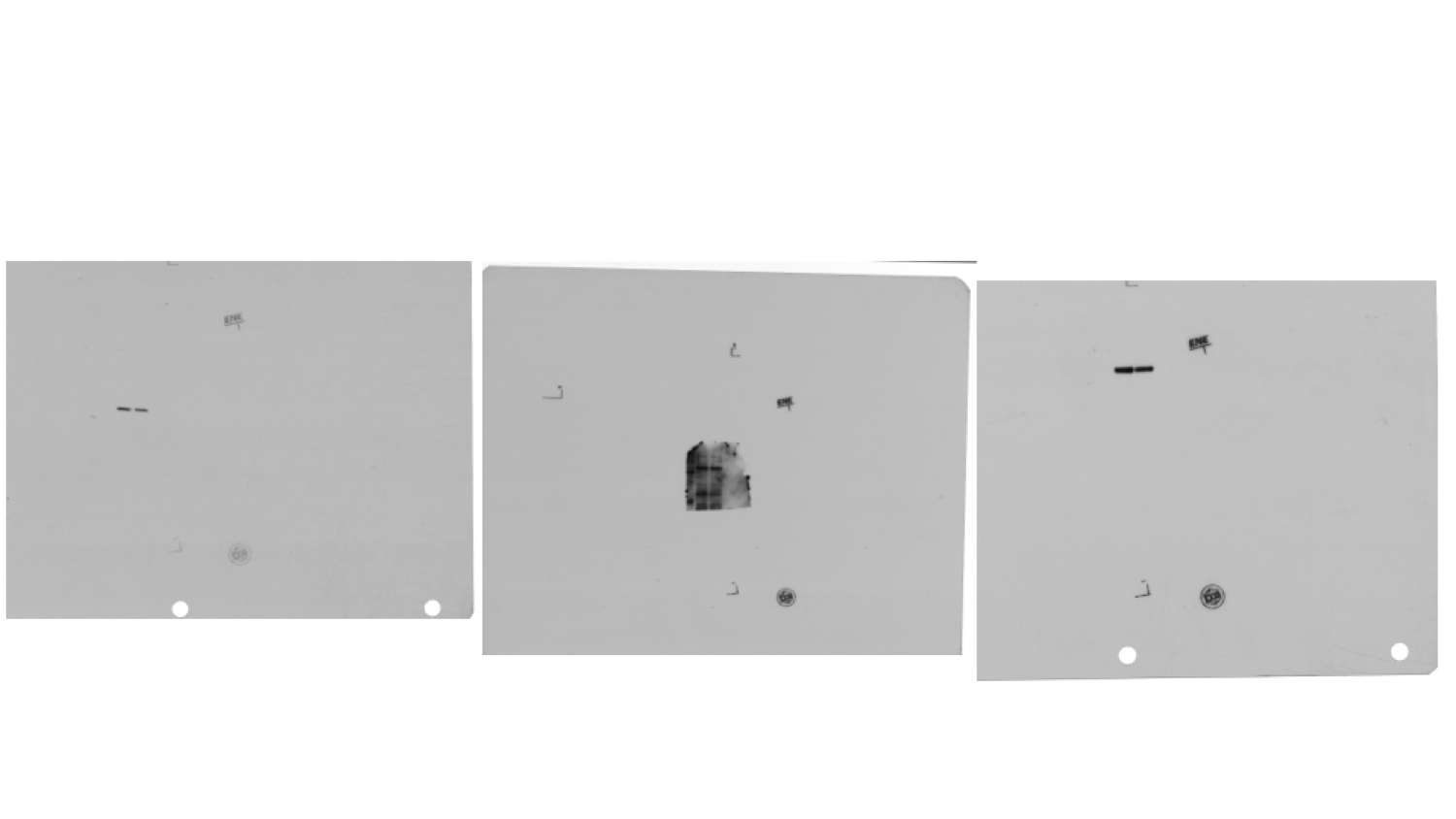

Supplement: Supplementary file 5 — Additional file 5: Figure 6A- unprocessed data. Western blots shown in figure 6A. Figure 6B- unprocessed data Western blots shown in figure 6B [file 12860_2020_282_MOESM5_ESM.zip › AdditionalFile6B.tiff]

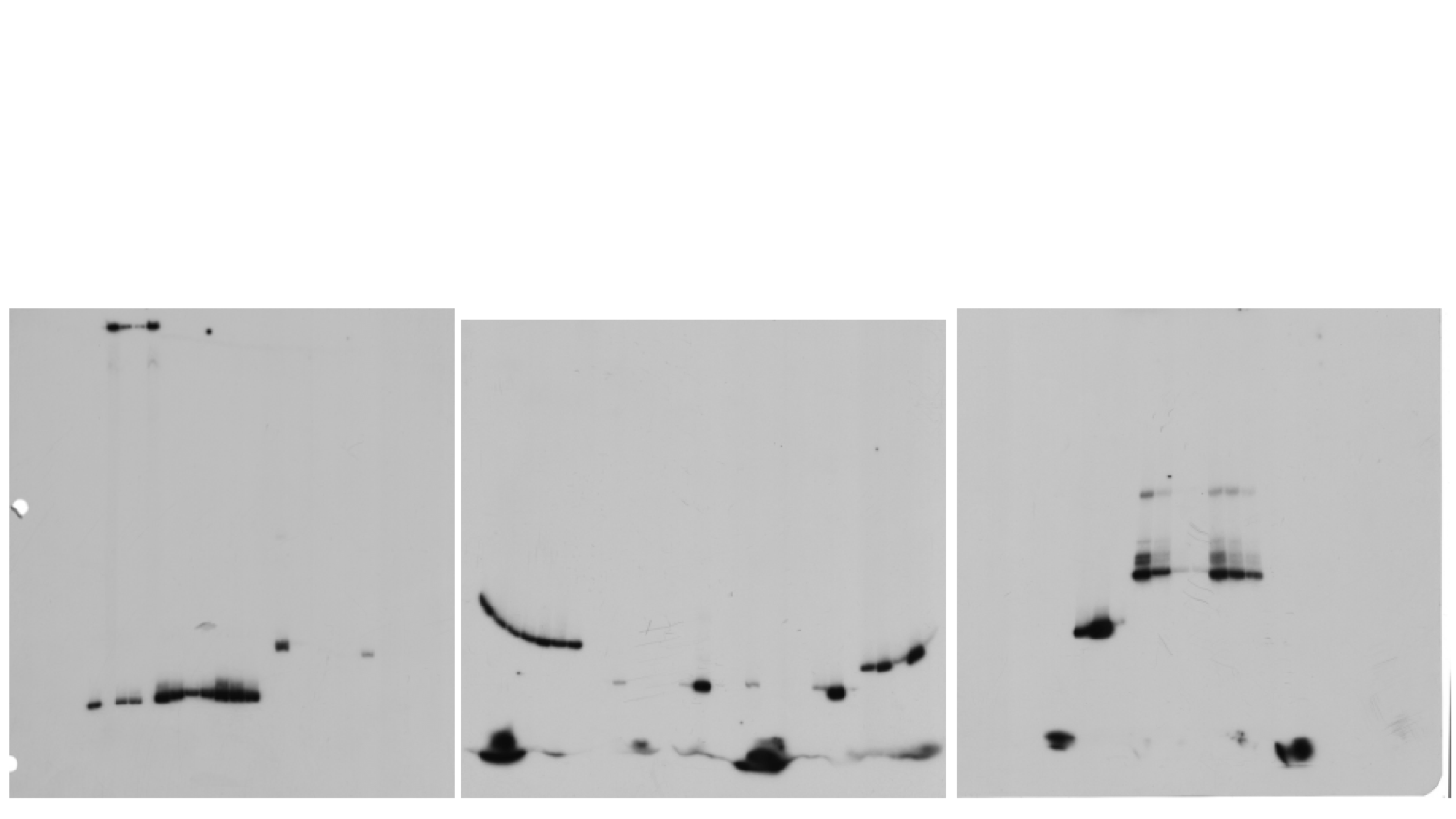

Supplement: Supplementary file 6 — Additional file 6: Figure 7A- unprocessed data. RT-PCR gels shown in figure 7A. Figure 7B- unprocessed data Western blots shown in figure 7B. Figure 7C- unprocessed data Western blots shown in figure 7C [file 12860_2020_282_MOESM6_ESM.zip › AdditionalFile7A.tiff]

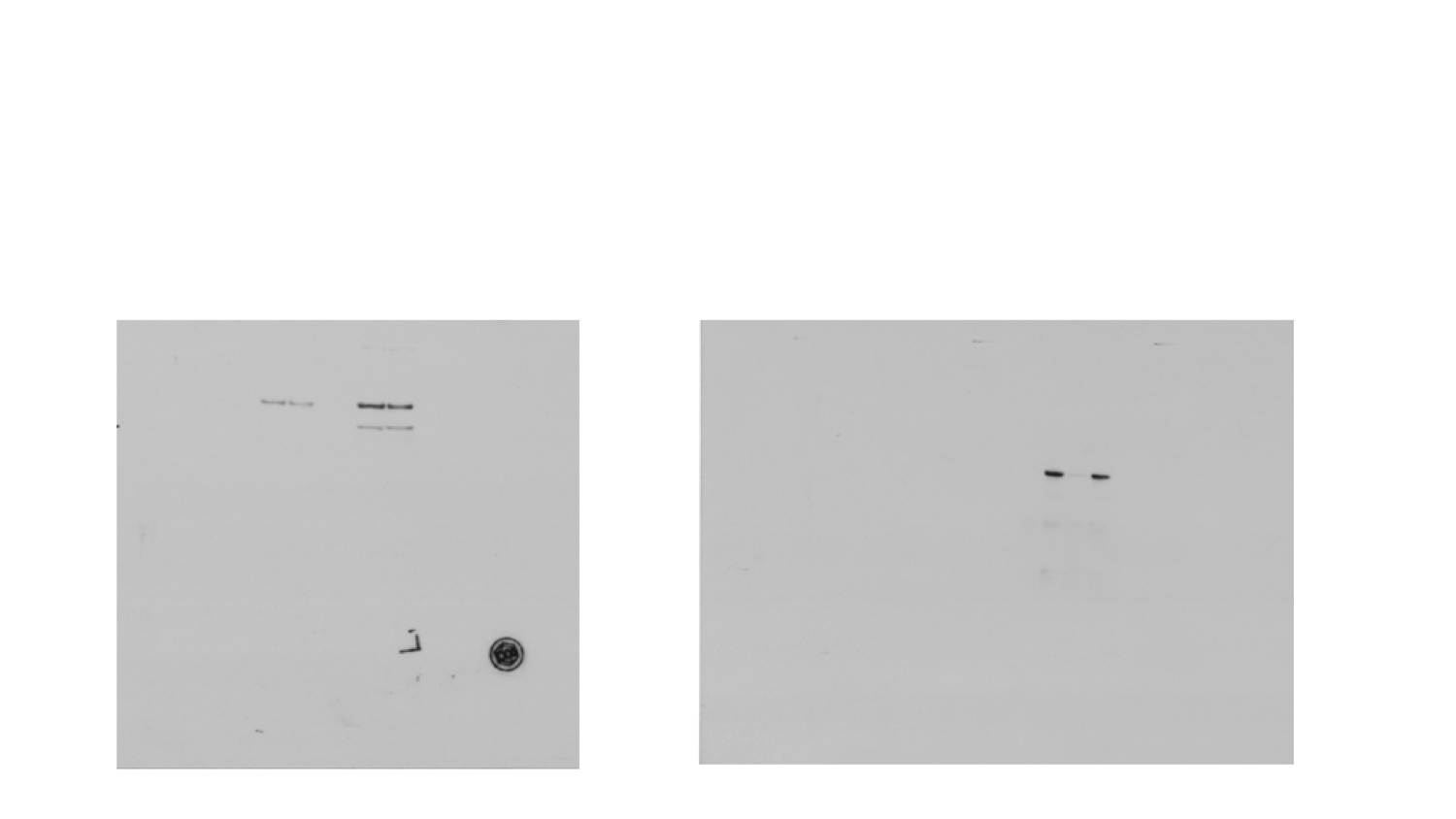

Supplement: Supplementary file 6 — Additional file 6: Figure 7A- unprocessed data. RT-PCR gels shown in figure 7A. Figure 7B- unprocessed data Western blots shown in figure 7B. Figure 7C- unprocessed data Western blots shown in figure 7C [file 12860_2020_282_MOESM6_ESM.zip › AdditionalFile7B.tiff]
